# Supplementary material for: Development of Sentinel LN Imaging with a Combination of HAase Based on a Comprehensive Analysis of the Intra-lymphatic Kinetics of LPs
Source: Mol Ther. 2020 Sep 6;29(1):225–35. doi: 10.1016/j.ymthe.2020.09.014 (PMC7791005; doi:10.1016/j.ymthe.2020.09.014)
Supplement: Document S1. Figures S1–S17 and Supplemental Materials and Methods [file mmc1.pdf]

YMTHE, Volume *29*

Supplemental Information

Development of Sentinel LN Imaging with a Combination of HAase Based on a Comprehensive Analysis of the Intra-lymphatic Kinetics of LPs

Masaki Gomi, Yu Sakurai, Takaharu Okada, Naoya Miura, Hiroki Tanaka, and Hidetaka Akita

Supplemental Information

Development of sentinel lymph node imaging with a combination of hyaluronidase based on a comprehensive analysis of the intra-lymphatic kinetics of liposomes

Masaki Gomi[1,‡], Yu Sakurai[1,‡,*], Takaharu Okada[2], Naoya Miura[1], Hiroki Tanaka[1], Hidetaka Akita[1,*].

AUTHOR ADDRESS

[1] Graduate School of Pharmaceutical Sciences, Chiba University, Chiba, Japan.

[2] RIKEN Center for Integrative Medical Sciences, Yokohama, Kanagawa, Japan

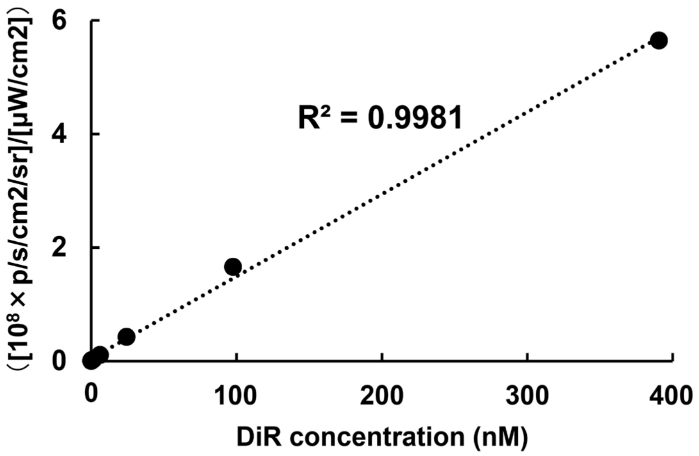

Supplemental Figure 1. Confirmation of the linearity of DiR fluorescence. The fluorescent intensity of known concentrations of DiR in the solution was detected by IVIS.

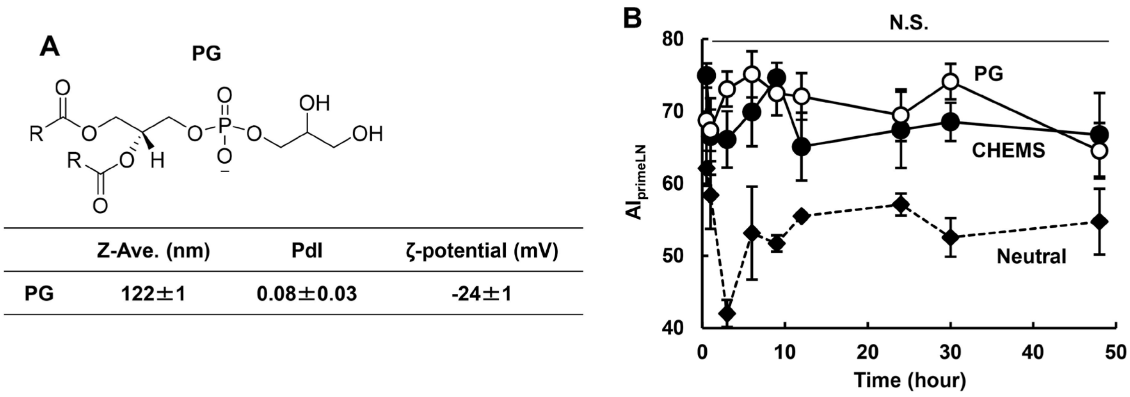

	Z-Ave. (nm)	PdI	ζ-potential (mV)
PG	122±1	0.08±0.03	-24±1

Supplemental Figure 2. The intra-lymphatic system distribution of LPs with other anionic lipids. A) The structure of PG is shown. R means -$C_{18}H_{33}$. LPs were characterized by DLS. B) Percentage of remaining in the primary LN was evaluated for periods of up to 48 hours. Data represents the mean ± SE (n=3). Student's t-test was carried out between PG and CHEMS LP at each time points. P-value > 0.05 was regarded as not statistically significant difference (N.S.).

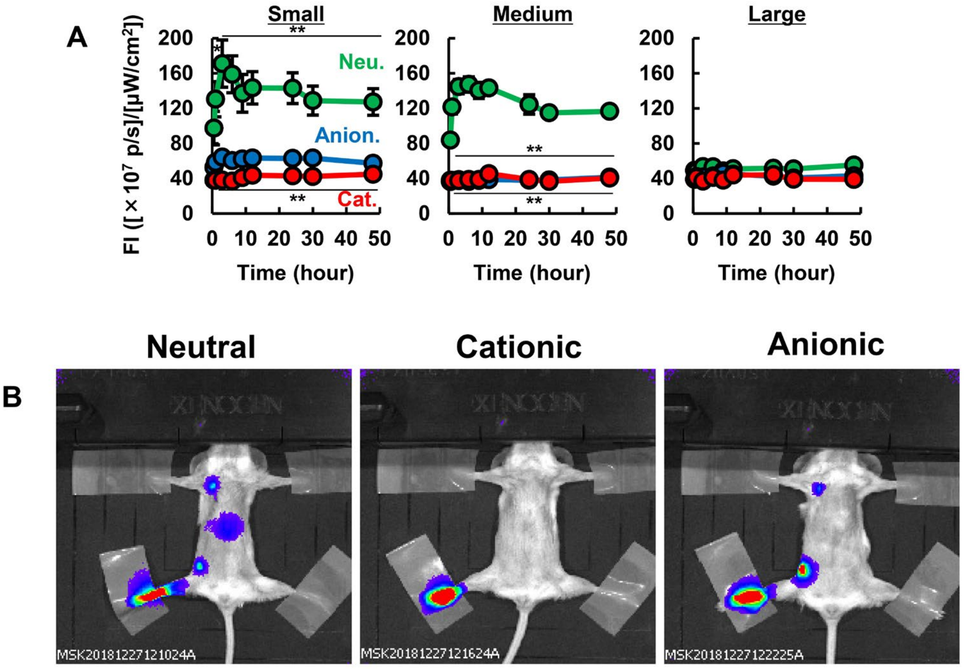

Supplemental Figure 3. Liver accumulation after the administration of LPs into lymphatic system. (A) Transport to the liver after the subcutaneous administration was measured by IVIS. The value represents the mean ± SE (n=3). At each time points, ANOVA was carried out, followed by Bonferroni test versus neutral LPs. N.S.: not statistically significant, *: P<0.05, **: P<0.01. (B) The representative images after the injection of medium-sized neutral, cationic and anionic LPs.

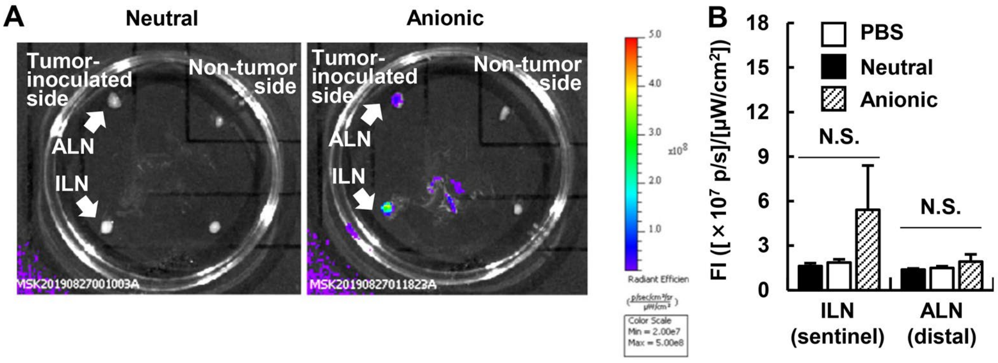

Supplemental Figure 4. Imaging of sentinel LNs in an orthotopic breast cancer model by medium-sized LPs. (A) Representative images of ILN (regarded as sentinel LN) and ALN (regarded as distal LN) of tumor-inoculated side (left) and not-inoculated side (right) 1 hour after the intratumoral injection of LPs. (B) The quantitative data from the (A) images. The value represents mean ± SE (n=3-5). At each time points, ANOVA was carried out, followed by Bonferroni test. N.S.: not statistically significant.

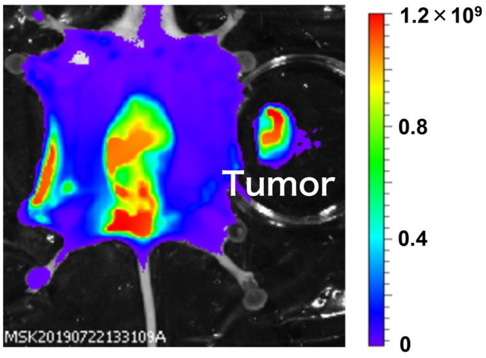

Supplemental Figure 5. The distribution of intratumoral injected indocyanine green (ICG). ICG was visualized by an IVIS system 1 hour after 20 μL of the ICG solution in PBS at 2.5 mg/mL was intratumorally administered.

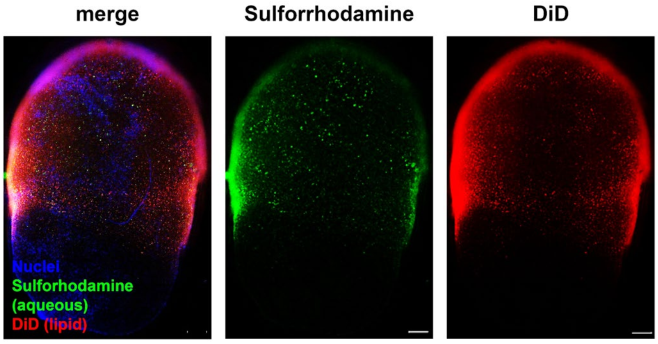

Supplemental Figure 6. Proof of LP delivery in intact form. Sulforhodamine was encapsulated into DiD-labeled anionic LPs. LN was observed 3 hours after the injection of LPs into LFM model. Blue, green and red dots show nuclei, sulforhodamine and DiD, respectively.

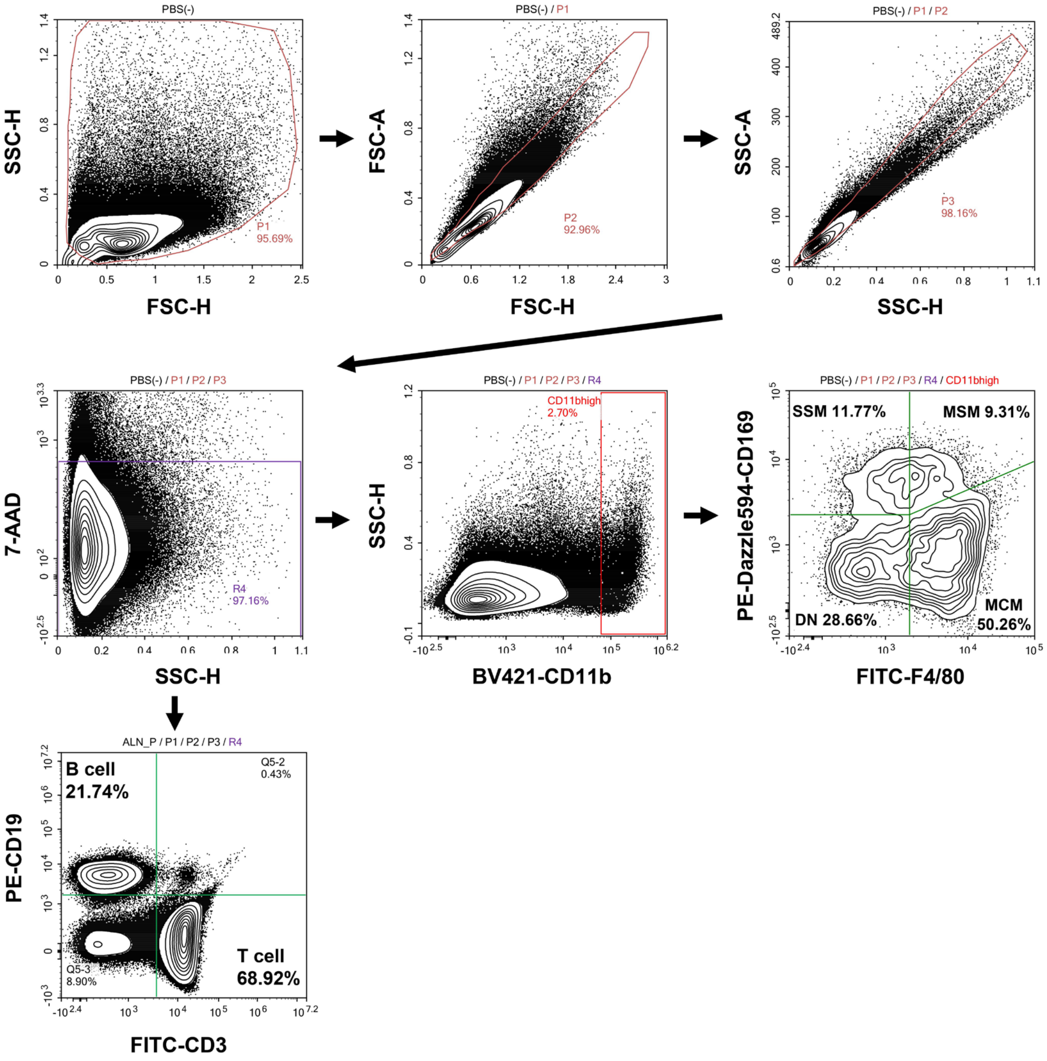

Supplemental Figure 7. A representative flow cytometer plot identifying SSM, MSM and MCM.

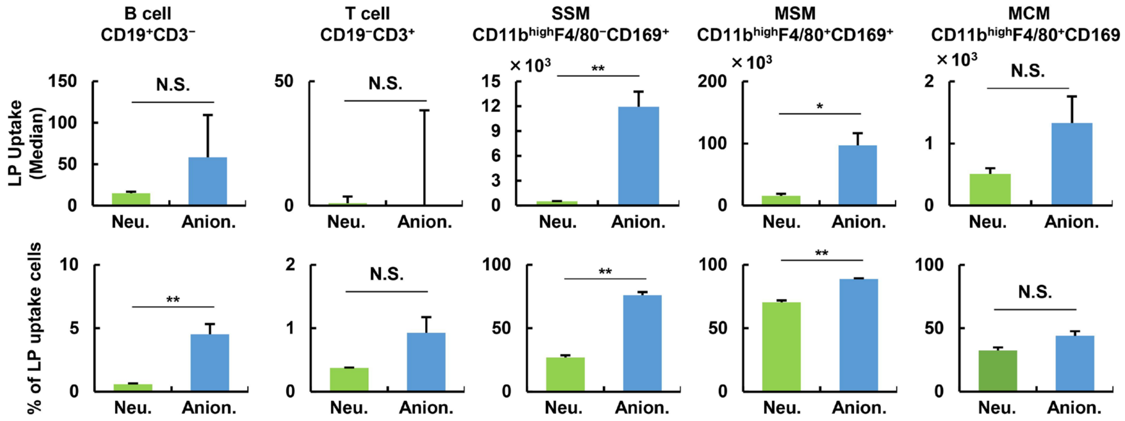

Supplemental Figure 8. Other quantitative data of cells with LPs. Median value of LPs uptake and percentages of the cells taking up LPs were analyzed. Data represent the mean ± SE (n=3). Student's t-test was performed for a pair-wise comparison. *: P<0.05, **: P<0.01.

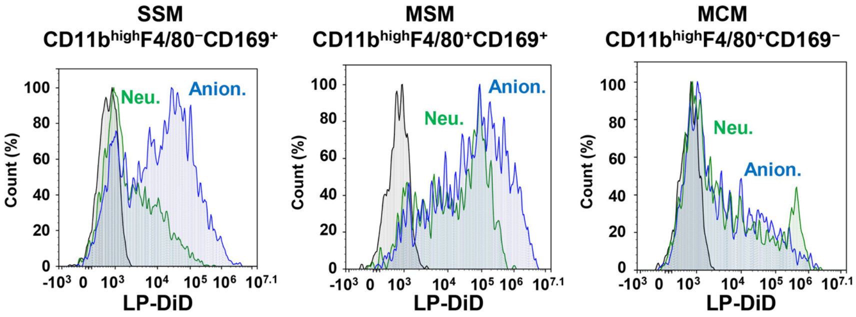

Supplemental Figure 9. The uptake of macrophages with normal (non-operated) mice. LNs were collected 2 hours after the LPs were administered into the tail base (BALC/c mice). Cells with LPs in the ILN were analyzed by flow cytometry.

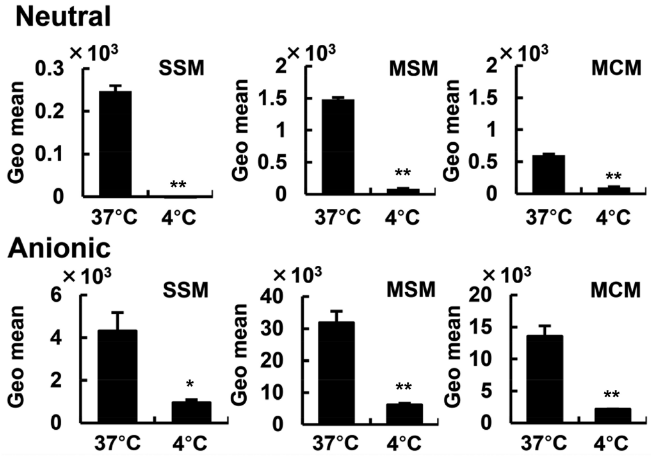

Supplemental Figure 10. The energy-dependent uptake of LPs by macrophages. LPs were incubated with cell suspensions isolated from ILN for 2 hours. Cells were analyzed by flow cytometry. Data represent the mean ± SE (n=3). Student's t-test was performed for a pair-wise comparison. *: P<0.05, **: P<0.01.

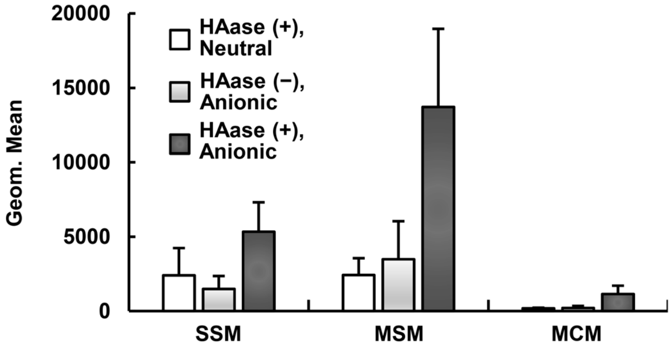

Supplemental Figure 11. Uptake of LPs by macrophages with tumor-bearing mice after intratumoral injection. Sentinel LNs were collected at 6 hours after the intratumoral injection of LPs. LNs were analyzed by flow cytometry. Data represents mean ± SE (n=3).

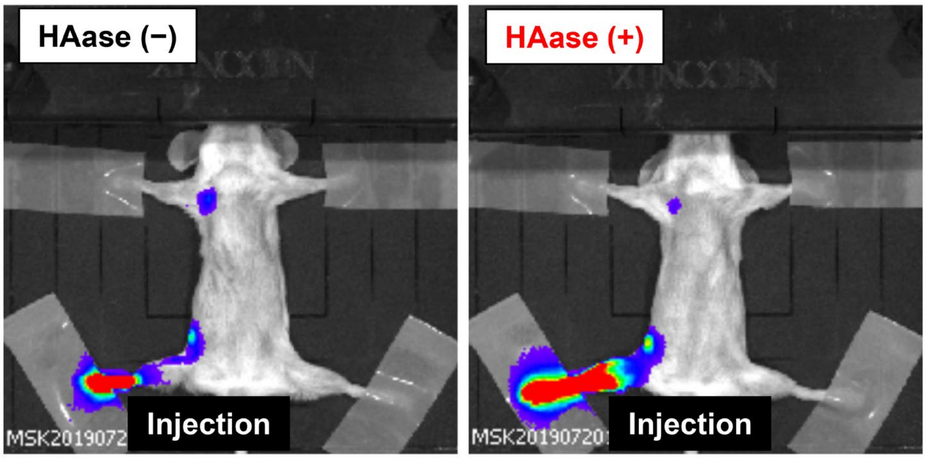

Supplemental Figure 12. The diffusion of LPs after the subcutaneous injection of anionic LPs into LFM mice. Mice were imaged by an IVIS system 30 min the injection.

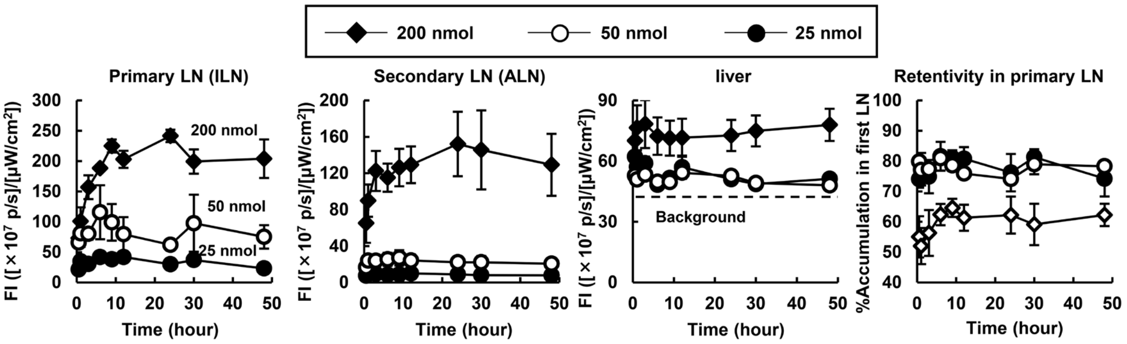

Supplemental Figure 13. Dose-dependent intra-lymphatic system distribution of medium sized anionic LPs. LPs were administered into the foot pad of LFM mice at a concentration of 25 – 200 nmol (100 nmol was used in the manuscript). LPs in the body were observed by an IVIS system for periods of up to 48 hours. Data represent the mean ± SE (n=3).

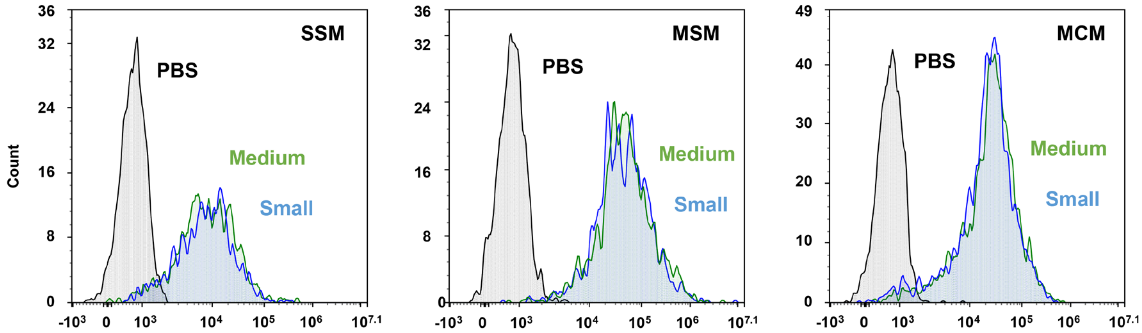

Supplemental Figure 14. The uptake small- and medium sized anionic LPs by macrophages. Small- and Medium-sized anionic LPs were incubated with a cell suspension from the LNs for 2 hours.

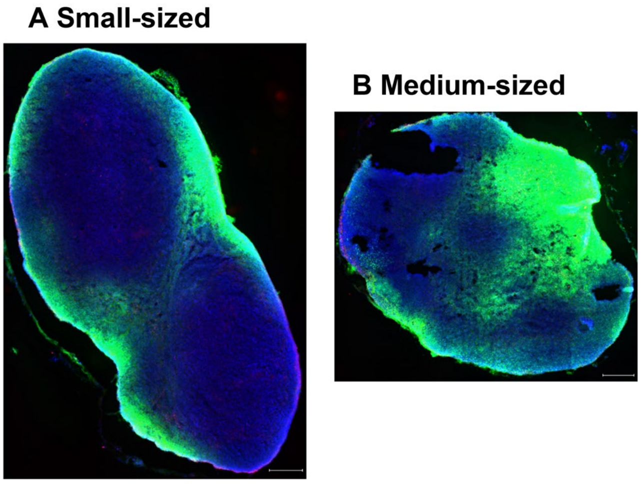

Supplemental Figure 15. The different distribution of anionic LPs with different sizes. A), B) After the subcutaneous injection of A) small-sized and B) medium sized anionic LPs, excised LNs were observed by microscopy 24 hours later. Blue and Green dots denote nuclei (Hoechst33342) and LPs (DiI), respectively.

Supplemental Figure 16. Antibodies and reagents used for the flow cytometry analysis.

Antibody/Reagent	clone/manufacture or catalog No.
CD16/32 FcγR blocking antibody	93
7-AAD	Biolegend, 420404
BV421-CD11b	M1/70
FITC-F4/80	BM8
PE/Dazzle594-CD169	3D6.112
FITC-CD3	17A2
PE-CD19	6D5

A Fluorescent intensity of each LPs

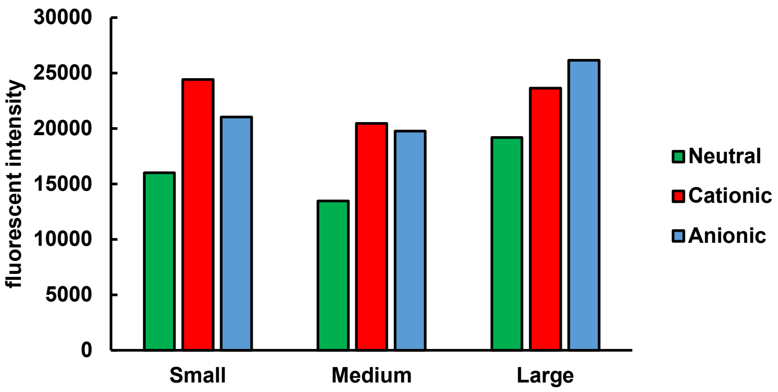

B Phospholipid concentration

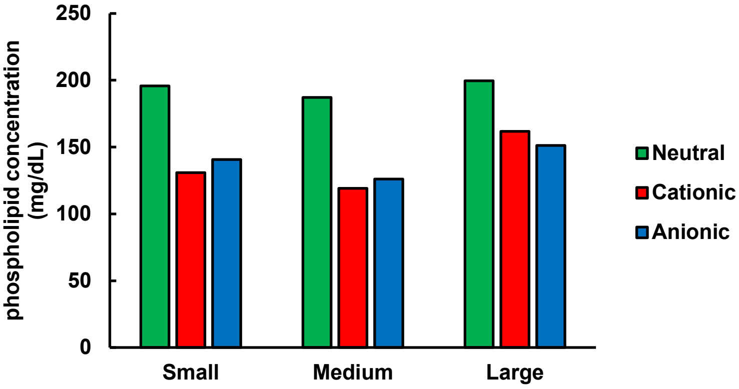

Supplemental Figure 17 The fluorescent intensity and phospholipid concentration of LPs used in Figure 2.

Supplemental materials & methods

Intravital imaging of popliteal LNs.

The entire procedure was performed in reference with the previous report.[1] CD11c-EYFP mice[2] were anesthetized with 1.5% isoflurane (Wako) in a stream of oxygen. Hair on the right hind legs was removed by an electric trimmer and the epilat cream (Kracie), and the epilated skin was cleaned and dried. Surgery was performed according to the previously described method (ref. 2), except that the leg holder assembly was modified as below such that intravital imaging of popliteal LNs could be carried out with an inverted microscope. The leg holder was assembled from a 35-mm Petri dish lid and two Eppendorf tube lids glued on the top of the Petri dish lid. The Eppendorf tube lids were placed approximately 6 mm apart from each other, both near the rim of the Petri dish lid. The right hind leg was glued between the Eppendorf tube lids with Aron alpha A "Sankyo" (Daiichi-Sankyo), with the back of the knee facing up. Skin flaps were made according to the previous report (ref. 2) and glued over the Eppendorf tube lids. The popliteal lymph node was micro-surgically exposed as described previously (ref. 2). The entire preparation was placed on an imaging platform (LEICA MATS, Tokai Hit) adjusted to 37 ℃, with the mouse body lying sideways, the leg holder placed upside down, and the exposed LN touching down on a drop of PBS on a coverslip which was glued with High Silicone II (Denken-Highdental) over the hole of the imaging platform. A catheter (C10SS-MTV1417P, Instech Laboratories) attached to a 1-ml syringe containing Sulforhodamine B-encapsulting LPs (40 nmol/ml lipid conc. in PBS) was inserted into the right hind footpad and immobilized by a vinyl tape. Image stacks of 45 z-sections at a x-y resolution of 0.16μm per pixel with a 1.0μm spacing between neighboring z-sections were acquired every 10 s by using the THUNDER 3D Live Imaging system (Leica Microsystems) equipped with an HC PLAPO CS2 40xW objective lens. EYFP signals were obtained by using a 470/24-nm LED and a 515/40-nm emission filter, and sulforhodamine signals were obtained by using a 550/15-nm LED and a 595/40-nm emission filter. During the time-lapse recordings, 50 μl of the LP suspension was injected into the footpad in 10~20 s. Obtained images were processed by the THUNDER Large Volume Computational Clearing algorithm (Leica Microsystems) using the following settings: Feature Scale 843 nm, Thunder Strength 94%, and Deconvolution Setting Auto.

References

1. Liou, H. L.; Myers, J. T.; Barkauskas, D. S.; Huang, A. Y., Intravital imaging of the mouse popliteal lymph node. *J Vis Exp* **2012,** (60).

2. Lindquist, R. L.; Shakhar, G.; Dudziak, D.; Wardemann, H.; Eisenreich, T.; Dustin, M. L.; Nussenzweig, M. C., Visualizing dendritic cell networks in vivo. *Nat Immunol* **2004,** *5* (12), 1243-50.
