# Supplementary material for: Development of Sentinel LN Imaging with a Combination of HAase Based on a Comprehensive Analysis of the Intra-lymphatic Kinetics of LPs
Source: Mol Ther. 2020 Sep 6;29(1):225–35. doi: 10.1016/j.ymthe.2020.09.014 (PMC7791005; doi:10.1016/j.ymthe.2020.09.014)
Supplement: Document S2. Article plus Supplemental Information [file mmc4.pdf]

Molecular Therapy
Original Article

Development of Sentinel LN Imaging with a Combination of HAase Based on a Comprehensive Analysis of the Intra-lymphatic Kinetics of LPs

Masaki Gomi,[1,3] Yu Sakurai,[1,3] Takaharu Okada,[2] Naoya Miura,[1] Hiroki Tanaka,[1] and Hidetaka Akita[1]

[1]Graduate School of Pharmaceutical Sciences, Chiba University, Chiba 264-0028, Japan; [2]RIKEN Center for Integrative Medical Sciences, Yokohama, Kanagawa, Japan

The sentinel lymph node (LN) is the first LN to which lymph fluid flows from tumor tissue. We identified the key parameters of liposomes (LPs) that affect their accumulation in regional (primary) LNs with minimum leakage to its connecting (secondary) LNs by a comprehensive analysis of the LN-to-LN trafficking of LPs with various surface charges and various sizes. We used a lymphatic flow-modified (LFM) mouse that allows for the chronological analysis of inguinal (primary) LN-to-axillary (secondary) LN at the body surface. As a result, the anionic medium-sized LPs (130 nm on average) exhibited the highest accumulation in the primary LNs. A mechanism-based analysis revealed that CD169-positive macrophages in LNs were the dominant cell population that captures anionic LPs. Sentinel LN imaging was also performed by the intratumoral injection of fluorescent medium-sized anionic LPs using a breast cancer orthotopic model. In comparison with the typically used contrast agent indocyanine green, the anionic LPs were detected in sentinel LNs with a high sensitivity. Additionally, the co-injection of hyaluronidase significantly improved the sensitivity of detection of the fluorescent LPs in sentinel LNs. In conclusion, medium-sized anionic LPs combined with hyaluronidase represents a potent strategy for investigating sentinel LNs.

INTRODUCTION

The lymphatic system is recognized as the third vasculature system next to arteries and veins. This system consists of lymphatic vessels and lymph nodes (LNs) that play a pivotal role in the lymphatic transport of antigens and immune cells, as well as for adjusting the body fluid balance.[1,2] LNs are the essential site where antigen-presenting cells present antigens to T cells and B cells to acquire adaptive immunity. Thus, delivering drugs directly to LNs would provide an opportunity to regulate adaptive immune responses. It should also be noted that LNs are also important in cancer therapy. Sentinel LNs are defined as the LN that cancer cells first gain access to. It has been reported that the removal of sentinel LNs with the infiltration of cancer cells significantly improves the overall survival of patients with breast cancer and melanomas.[3,4] Real-time monitoring through near-infrared fluorescence is currently expected to be a viable method for the detection of sentinel LNs in clinic. Thus, the highly sensitive detection of sentinel LNs in surgical patients is an important technology for their efficient removal. Fluorescent contrast agents such as indocyanine green (ICG) and radioactive probe are currently used to accomplish this.[5] One of the major obstacles to sentinel LN imaging is the low sentinel/non-sentinel LN fluorescent ratio and the diffusion of fluorescence dyes from the sentinel LN to surrounding tissues and its connecting LNs.[6,7] Recent papers with breast cancer patients suggest that ICG promptly flows to other tissues within a few minutes because the hydrodynamic diameter of ICG is <1 nm.[8–10] This fast spread to non-sentinel LNs would lead to undesirable resection of normal LNs. Additionally, an operation time is severely restricted because of this rapid diffusion of the ICG.[11]

To address this issue, nanoparticles would be a suitable agent for use in sentinel LN imaging because of preferable physicochemical properties. Small, subcutaneously administered compounds preferably infiltrate the blood vasculature. In contrast, nanoparticles with sizes of ~20–100 nm are selectively taken up by the lymphatic system, because such nanoparticles cannot overcome the blood-endothelial barrier.[12–15] Accordingly, controlling the distribution of the nanoparticle in the intra-lymphatic system after it enters the lymphatic system would be a promising strategy for addressing the issue of lymphatic diseases. In a previous study, we focused on the transport of nanoparticles into the lymphatic system from the injection site and the accumulation of regional LNs (typically popliteal and iliac LNs). However, the quantitative pharmacokinetics of the movement of nanoparticles from the primary LN to the connecting LN (secondary LN) is not well understood. It should be noted that the development of a nanoparticle with extensive retention in primary LNs would be a promising technology for the identification of primary LNs.

We recently established a lymphatic flow-modified (LFM) mouse model that could be used in non-invasive studies of LN-to-LN trafficking.[16] In the case of normal mice, the LPs that are subcutaneously injected into the foot pad largely move to the popliteal LN and are

Received 17 May 2020; accepted 3 September 2020;
https://doi.org/10.1016/j.ymthe.2020.09.014.

[3]These authors contributed equally to this work.

Correspondence: Hidetaka Akita, Graduate School of Pharmaceutical Sciences, Chiba University, Inohana 1-8-1, Chuo-ku, Chiba 264-0028, Japan.
E-mail: akitahide@chiba-u.jp
Correspondence: Yu Sakurai, Graduate School of Pharmaceutical Sciences, Chiba University, Inohana 1-8-1, Chuo-ku, Chiba 264-0028, Japan.
E-mail: yu_sakurai@chiba-u.jp

This is an open access article under the CC BY license (http://creativecommons.org/licenses/by/4.0/).

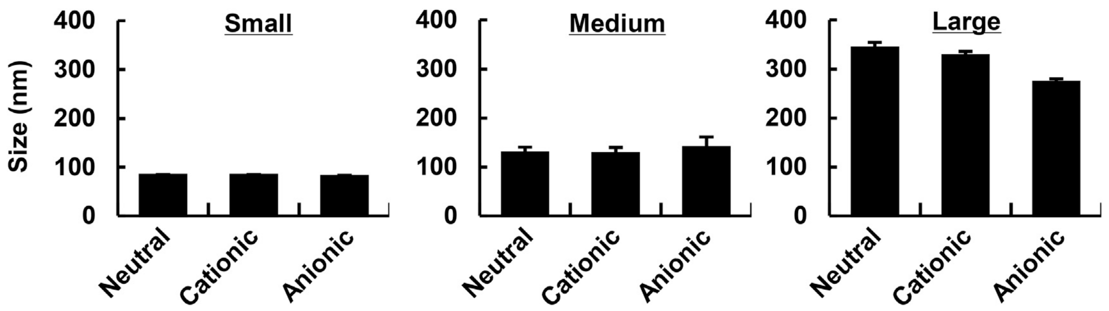

PdI	Small	Medium	Large
Neutral	0.06 ± 0.01	0.06 ± 0.01	0.08 ± 0.02
Cationic	0.09 ± 0.02	0.05 ± 0.03	0.17 ± 0.09
Anionic	0.18 ± 0.03	0.22 ± 0.01	0.24 ± 0.01

ζ-potential (mV)	Small	Medium	Large
Neutral	−0.3 ± 0.6	0.6 ± 0.5	0.0 ± 0.5
Cationic	25.7 ± 1.6	26.4 ± 0.2	32.0 ± 0.8
Anionic	−28.3 ± 0.7	−32.0 ± 6.2	−36.8 ± 0.2

Figure 1. Characterization of LPs

LPs were characterized by dynamic light scattering. Data represent the mean ± standard deviation (n = 3).

then drained into iliac LNs, which are located deep inside of the body. Thus, the continuous observation of nanoparticles in the iliac LN without sacrifice is a difficult task. In contrast, in the LFM mouse model, in which popliteal LNs had been removed, lymphatic flow from the foot pad to the inguinal LN (ILN) and its connecting axillary LN (ALN) are induced. Because these LNs are located in fat tissue under the skin surface, fluorescent-labeled nanoparticles in these LNs could be readily observed by an *in vivo* imaging system (IVIS).

In the present study, we report on the first comprehensive analysis of the intra-lymphatic pharmacokinetics of liposomes (LPs) with various sizes and ζ-potentials, with an extensive focus on the LN-to-LN tracking, as well as a drainage time profile into the lymphatic system from the injection site. As described below, our analyses revealed that LPs with specified physicochemical properties accumulated extensively in the primary LNs. The second goal was to identify the mechanism and/or driving force for this preferred accumulation of primary LNs. Finally, we report on the application of this LP for use in the sensing of sentinel LNs with an aid of hyaluronidase (HAase).

RESULTS

Optimization of LPs Physicochemical Properties Using the LFM Model

Neutral, anionic, and cationic LPs in various sizes were prepared with an Extruder. The surface charge of the LPs was controlled by replacing the neutral cholesterol (chol) in the particles with cationic and anionic chol derivatives (chol hydrochloride [DC-chol], cholesteryl hemisuccinate [CHEMS]). The physicochemical properties of the prepared LPs are summarized in Figure 1. In the following study, the small, mid-sized, and large LPs are defined as those with sizes of approximately (approx.) 70, 130, and >300 nm, respectively. The ζ-potentials of the positively and negatively charged LPs were also controlled at ~+25–30 mV and ~−28 to 37 mV regardless of their size. The LFM model mouse was prepared by removing popliteal LNs by ligating the efferent and afferent lymphatic vessels, as well as marginal veins and the feeding blood vessels (Figure 2A). After administering each LP into the foot pad, the fluorescence intensities in the ILN (primary) and ALN (secondary) were evaluated by IVIS imaging for periods of up to 48 h (Figures 2B and 2C). All of the following study was conducted in the range in which the fluorescence signal was not saturated, and the fluorescence intensity (FI) was linearly increased against the amount of fluorescent dye (Figure S1). In PBS-treated mice, autofluorescence in the LNs was less than 3.5×10^7 ([photon/s]/[μW/cm^2]). The findings clearly showed that, in the case of the large-sized LPs, their drainage in ILNs and ALNs was quite poor regardless of the surface charge of LPs. These results are consistent with previous reports showing that particles with sizes of less than 170 nm are able to enter the lymphatic system.[17] The second interesting finding was that in the case of small- and middle-sized LPs, the accumulation of the cationic LPs in primary LNs (ILNs)

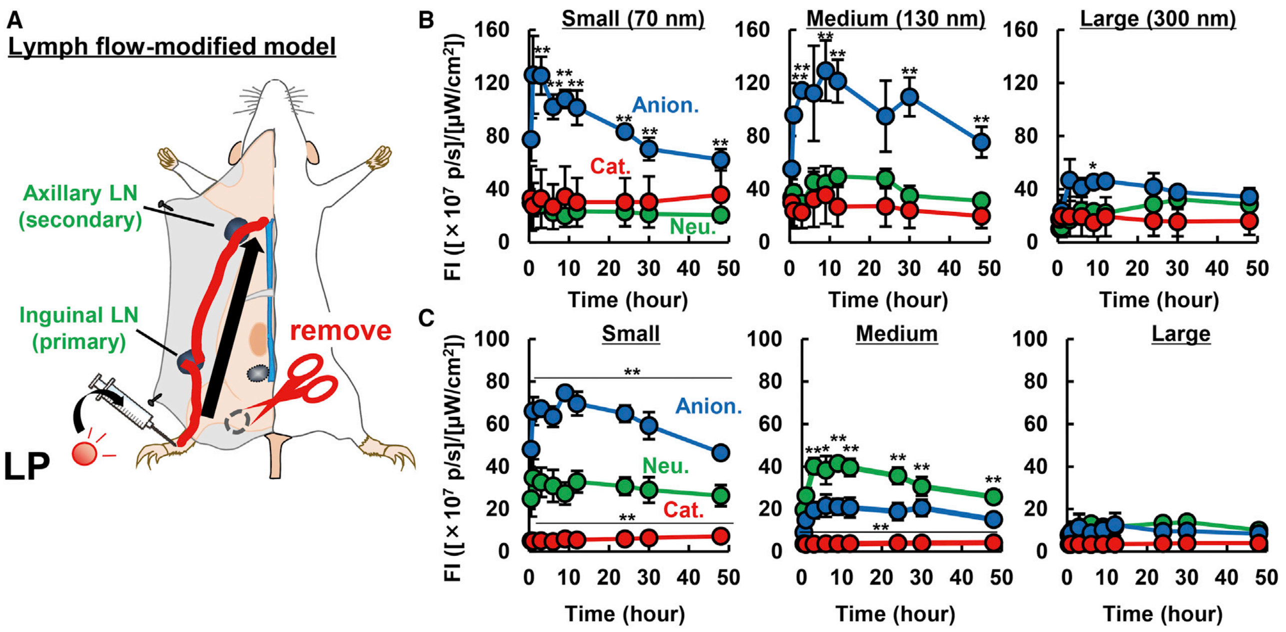

Figure 2. Comprehensive Analysis of Intra-lymphatic System Kinetics of LPs

(A) A schematic diagram of the animal model (LFM model) used in this study. Prior to the experiment, the popliteal LN was removed by ligating the vessels to induce a new lymph flow from the foot pad to the inguinal LNs. (B and C) The accumulation of (B) primary LNs (inguinal LNs) and (C) secondary LNs (axillary LNs) of anionic (Anion.), cationic (Cat.), and neutral (Neu.) LPs with different sizes (70, 130, and 300 nm). The value represents the mean ± standard error (SE) (n = 3). At each time point, ANOVA was carried out, followed by Bonferroni test versus neutral LPs. *p < 0.05, **p < 0.01. N.S., not statistically significant.

was less than that for the neutral LPs, and moreover that of anionic LPs was significantly higher than the corresponding values for the neutral ones. The most significant finding was the difference in the LN-to-LN trafficking of neutral and negative LPs. Both small- and medium-sized anionic LPs accumulated more preferably compared with neutral LPs in the primary LN. Of note, in the case of small LPs, the accumulation of LPs in the secondary LN (ALN) was higher for anionic LPs in comparison with the neutral species, in parallel with the preferred accumulation of anionic LPs in primary LNs (ILNs).

In contrast, in the case of middle-sized LPs, the accumulation of anionic LPs in secondary LNs (ALN) was significantly less than that for neutral LPs, whereas the drainage of anionic LPs in the primary LN (ILN) were higher than that for neutral LPs. These results suggest that controlling the size of LPs in the medium range (approx. 130 nm in size) and a negative ζ-potential (approx. −20 mV) result in an LP that showed the highest retention in the primary LN. This inference is supported by the calculated primary LN-accumulation index ($AI_{primeLN}$) that is denoted as the accumulation of an LP in the primary LN divided by the total lymphatic drainage (total LPs in primary and secondary LNs) (Figure 3). In the calculation, cationic LPs were excluded because the lymphatic drainage itself was poor. As a result, medium-sized anionic LPs exhibited the highest value (approx. 86%). In contrast, the corresponding value of neutral LPs was approx. 50% regardless of size. To confirm whether this primary LN-preferred accumulation was also achieved when other types of anionic lipids were used, we calculated the $AI_{primeLN}$ values of anionic LPs that were composed of EPC and anionic lipids, including dioleoyl-sn-glycerophosphoglycerol (DOPG) (Figure S2). As a result, high $AI_{primeLN}$ values above that for neutral LPs were also obtained for DOPG-containing LPs, indicating that the LPs with anionic surface would preferably stay in the primary LN. Thus, it is plausible that physicochemical properties, but not the type of anionic lipid structure, are a key factor for the accumulation of LPs in primary LNs. The liver accumulation of each of the LPs was evaluated, as measured by leakage from the injection site and/or transfer to the circulation after passage through the lymphatic trafficking system (Figure S3). As a result, substantial levels of neutral LPs accumulated in the liver tissue, whereas cationic and anionic LPs did not. Based on the screening of LPs with appropriate physicochemical properties using LFM model mice, we identified medium-sized and anionic surface as the most appropriate physicochemical properties for sentinel LN imaging. Accordingly, we performed sentinel LN imaging using medium-sized anionic LPs with the orthotopic breast cancer model. As mentioned in the Introduction, retention in the LN to which lymphatic fluid from tumor tissue first flows would be important to circumvent false-positive sentinel LN detection. Medium-sized anionic and neutral LPs were intratumorally administered, and the fluorescence of LPs in ILNs (regarded as sentinel LN) and ALN (regarded as distal LN) was then observed (Figure S4A). Fluorescence was detected in the sentinel LNs in the case of medium-sized anionic LPs (5.8×10^7 [(photon/sec)/(µW/cm^2)]), but not medium-sized neutral LPs (1.8×10^7 [(photon/sec)/(µW/cm^2)] under detection limit) 6 h after the injection of labeled LPs (Figure S4B). Of note, the trafficking to

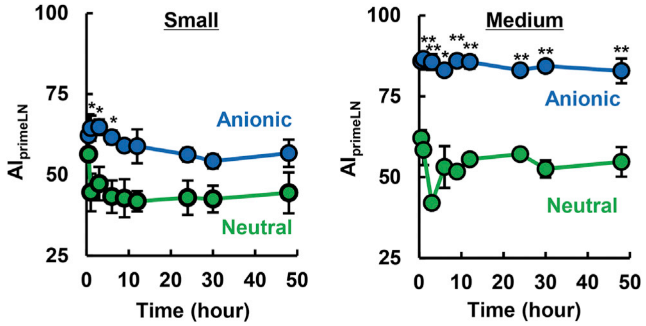

Figure 3. Calculated Selectivity for the Primary LN after Subcutaneous Injection

The selectivity in the primary LN was calculated by dividing the fluorescence intensity in the primary LN by the total fluorescence in the primary and secondary LNs. The value represents the mean ± SE (n = 3). At each time point, the Student's t test was performed between anionic and neutral LPs. *p < 0.05, **p < 0.01.

the ALN was under the detection limit. However, the difference is not statistically significant. The results suggest that improvement in the methodology will be needed for the further development of sentinel LN detection technology. In contrast, ICG, which is clinically used in the imaging of sentinel LN for the intraoperative biopsies, failed to specifically detect sentinel LNs because ICG was widely diffused in the entire body after intratumoral injection even just 1 h after the injection as mentioned in the Introduction (Figure S5). As mentioned in the Introduction, ICG promptly spread, and thus this rapid spread of ICG made it difficult to identify sentinel LNs. The above collective findings indicate that medium-sized anionic LPs are more suitable probes for use in examining sentinel LNs than currently used imaging agents.

Investigation of the Reason for the Preferable Accumulation of Medium-Sized Anionic LPs in the Primary LN

We next examined the mechanism responsible for the primary LN-preferred accumulation of medium-sized anionic LPs. Previous reports showed that anionically charged nanoparticles preferably entered the lymphatic system by charge repulsion between the anionic surface and the extracellular matrix (ECM).[18,19] Thus, the lymphatic trafficking of anionic LPs was evaluated after the enzymatic digestion of hyaluronan (HA) by HAase, a dominant anionic stroma in the ECM,[20] based on the assumption that the HAase treatment would lessen the degree of electrostatic repulsion between the anionic LP and the ECM, and that this would consequently result in a reduction in lymphatic drainage. Against our expectation, the co-administration of HAase resulted in a moderate increase in infiltration into the lymphatic system (approx. 1.5-fold for anionic LP, 2.0-fold for neutral LP) and the accumulation of both neutral and anionic LPs in primary LNs (Figures 4A and 4B). Although the drainage of both neutral and negative LPs tended to be increased by the HAase treatment, the higher $AI_{primeLN}$ values for the anionic LPs were retained (Figure 4C). Thus, an active mechanism is associated with the extensive retention of anionic LPs in primary LNs and the avoidance in capturing the neutral LPs. The hepatic accumulation of anionic LPs remained low even after the HAase treatment, whereas that of neutral LPs tended to increase in parallel with its distribution to the ILN and ALN (Figure 4D).

To better understand the mechanism responsible for the retention of anionic LPs in primary LNs, we compared the localization of medium-sized neutral and anionic LPs in primary LNs. Neutral LPs accumulated adjacent to afferent and efferent lymphatic vessels (Figure 5A). On the other hand, medium-sized anionic LPs diffused throughout the subcapsular sinus and medullary sinus (Figure 5B). To ask whether the LPs accumulated in an intact form, we determined the distribution of the LPs that were labeled with an aqueous marker sulforhodamine and a lipidic marker DiD (2-[5-(1,3-Dihydro-3,3-dimethyl-1-octadecyl-2H-indol-2-ylidene)-1,3-pentadien-1-yl]-3,3-dimethyl-1-octadecyl-3H-indolium perchlorate). As a result, the fluorescent signals of sulforhodamine and DiD were co-localized, indicating that LPs did not aggregate, nor did the free fluorescence per se accumulate (Figure S6). A fraction of the anionic LPs was co-localized with $CD11b^+$ macrophages in the subcapsular sinus and medullary sinus. We speculated that anionic LPs would be taken up by $CD11b^+$ macrophages in these areas, and consequently medium-sized anionic LPs showed higher retention than neutral ones. This speculation is consistent with the previous observation using transmission electron microscopy showing that gold colloid-encapsulated neutral and anionic LPs are taken up by macrophages.[21] To identify the cell fractions that are responsible for the uptake of LPs more quantitatively, we collected primary LNs (ILNs) at 24 h after the administration of the LPs and prepared a cell suspension for flow cytometry. In this analysis, the uptake of neutral and anionic LPs by T cells ($CD3^+$, $CD19^-$), B cell ($CD3^-$, $CD19^+$), subcapsular sinus macrophages (SSMs; $CD11b^{high}$, $F4/80^-$, $CD169^+$), medullary sinus macrophages (MSMs; $CD11b^{high}$, $F4/80^-$, $CD169^+$), and medullary cord macrophages (MCMs; $CD11b^{high}$, $F4/80^+$, $CD169^-$) was evaluated (Figure S7). T cells and B cells were selected because they occupied a large cell population in the LN. Uptake by dendritic cells is not currently evaluated because LPs did not localize in the paracortex where dendritic cells locate. As a result, no uptake of neutral or anionic LPs in T cells could be detected. Similarly, in the case of B cells, the uptake of neutral LPs was under the detection limit, while a small fraction of the negative LPs was taken up. Thus, although the uptake of the negative LPs was statistically higher than that for neutral ones, the little uptake by B cells cannot explain the retention of anionic LPs in primary LNs, despite that these are the dominant cells in LNs (Figure 6A). In contrast, a significantly higher uptake of the anionic LP was observed in the SSMs and MSMs, but not the MCMs (Figure 6A; Figure S8). The anionic LPs-preferred uptake in the SSMs and MSMs was also reproduced in normal BALB/c mice (not operated) after the subcutaneous injection of LPs into the tail base (Figure S9). Such a rapid and efficient accumulation of anionic LPs in comparison with the neutral ones was also supported by intravital confocal laser microscopy observations (Videos S1 and S2). These sets of experiments indicate that the preferred uptake of LPs to the SSMs and MSMs is responsible for the extensive retention of anionic LPs in primary LNs in the LFM model mice.

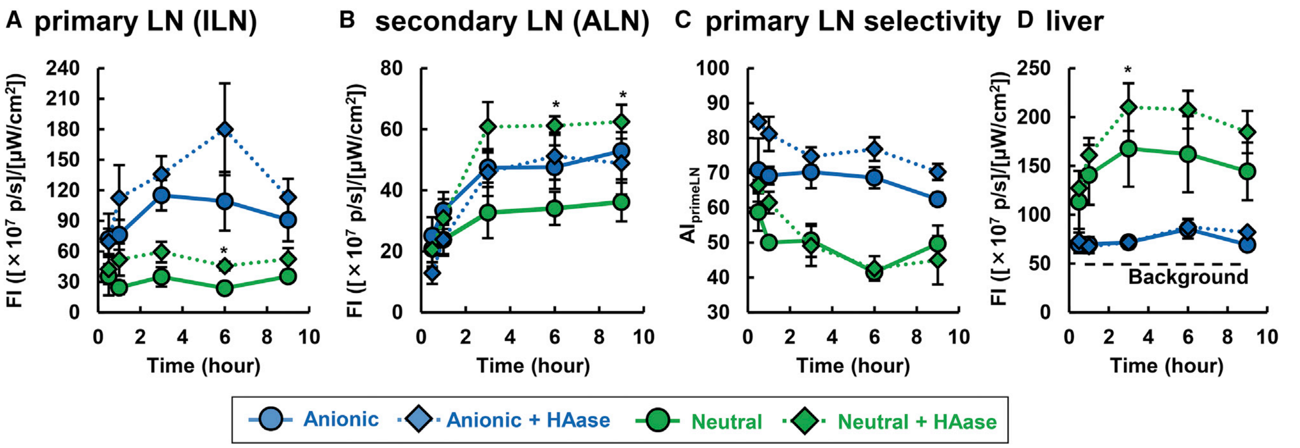

Figure 4. Effect of HAase on the Intra-lymphatic System Distribution of LPs

(A and B) At the indicated time points, the accumulation of LPs in (A) the primary LN and (B) the secondary LN was evaluated. (C) The selectivity for the primary LN was calculated in terms of $AI_{primeLN}$. (D) Accumulation in the liver after the subcutaneous administration of LPs, indicating the extent of leakage from the lymphatic system. The value represents the mean ± SE (n = 3). At each time point, the Student's t test was performed between HAase (–) and (+). $^{*}p < 0.05$.

Because CD169 (sialic acid-binding immunoglobulin-type lectins: Siglec-1) is expressed in the SSMs and MSMs as a typical marker, but not in the MCMs, and it is known to be involved in the uptake of oxidized low-density lipoprotein (LDL) in cooperation with the scavenger receptor B1 (SR-B1), we assumed that CD169 might play a role in the uptake of anionic LPs, based on an analogy in which LPs are taken up via LDL receptors in the body.[22–24] To investigate this hypothesis, we incubated LPs with cell suspensions that were preliminarily prepared from the isolated LNs. As a result, anionic LP-preferred uptake in preference to neutral species was observed in all subtypes of macrophages, even in the CD169-negative MCMs (Figure 7). This result suggests that CD169 itself does not function as a receptor for anionic LPs. Rather, the intrinsically high phagocytic activity against anionic LPs was a common feature among all of the macrophage fractions. However, when the LPs were exposed to a LN cell suspension at 4°C, the uptake of neutral LPs by the SSMs and MSMs was nearly completely suppressed because of the inhibition of energy-dependent phagocytosis, while a significant portion of the cellular association remained in the anionic LPs (Figure S10).

Sentinel LN Detection by Combining Medium-Sized Anionic LPs with HAase

As mentioned above (Figure 4), the co-administration of HAase induced the lymphatic drainage of LPs. ECMs such as collagen, elastin, and HA also hamper the intra-tumoral distribution of nanoparticles by steric hindrance.[25,26] Previous reports have suggested that HA was a major component for the obstruction in the diffusion of nanoparticles rather than collagen.[27,28] These data prompted us to hypothesize that the use of a combination of anionic LPs and HAase could improve the sensitivity of sentinel LN. As expected, a HAase treatment improved the drainage of both neutral and anionic medium-sized LPs (Figures 8A and 8B). To assess the specificity for the primary LN of anionic and neutral LPs, we calculated the $AI_{primeLN}$ values from imaging data from tumor-bearing mice (Figure 8C). As a consequence, regarding the neutral LPs, they were detected in both sentinel and distal LNs. In contrast, anionic LPs exhibited a better selectivity for sentinel LNs with less fluorescence signal intensity in distal LNs even 6 h after the injection of LPs. Meanwhile, intratumorally injected free ICG rapidly diffused from the sentinel LN (Figure S5), indicating that there is not much time left for resection of sentinel LN after the dye injection. In contrast with ICG, sentinel LN imaging with anionic medium-sized LPs could give a time extension in anatomical resection of sentinel LNs. When the tumor tissue was treated with HAase, the uptake by SSMs and MSMs in sentinel LNs was increased as demonstrated before in the primary LN with the LFM model (Figure S11).

DISCUSSION

Our comprehensive evaluation suggests that anionic LPs tend to be retained in the lymphatic system, especially in the primary LN, whereas neutral LPs circulated in the lymphatic system without capture by the LNs, and subsequently are transported to the blood circulation via the subclavian vein. Cationic LPs were not seen in either the primary LN or the secondary LN (Figures 2B and 2C). These data are also in agreement with the currently accepted concept that the lymphatic drainage of cationic LPs would be hampered by the electrostatic interactions with the negatively charged ECM and vice versa in anionic LPs via electrostatic repulsion with it (Figure 2B).[12] Several studies have indicated that cationic nanoparticles failed to enter the lymphatic system.[29–31] The authors also inferred that this retention in the subcutis might be a result of electrostatic interactions between nanoparticles and anionic ECMs, such as glycosaminoglycans and proteoglycans. However, HAase treatment enhanced lymphatic transport of anionic LPs (Figure 4). This result suggests that electrostatic repulsion, at least by HA, was not a dominant driving force for the accelerated infiltration of anionic LPs into the lymphatic system. Rather, it appears that the ECM might restrict the entrance of LPs into the lymphatic system by entrapping the

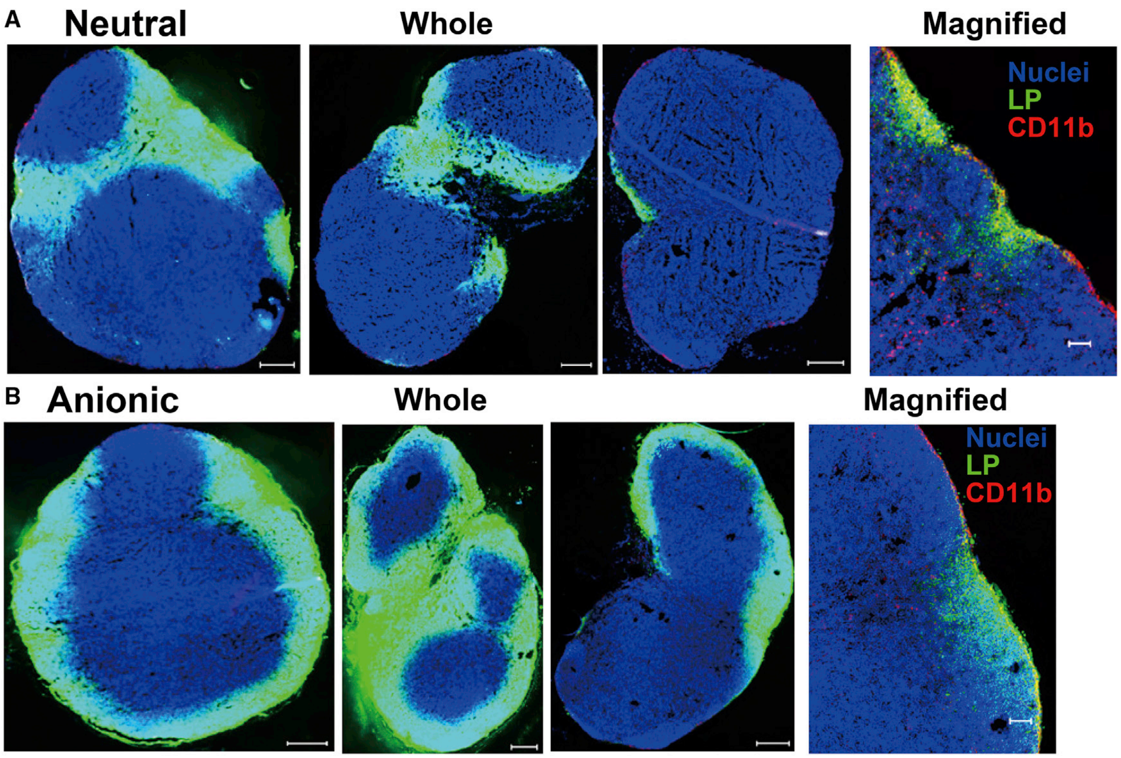

Figure 5. Localization of LPs in Primary LNs

(A and B) LNs were stained with Hoechst 33342 (nuclei), DiI (LPs), and CD11b (macrophages) 24 h after the subcutaneous injection of (A) neutral or (B) anionic LPs into LFM mice. Scale bars: 200 μm (left panels); 50 μm (right panels).

nanoparticle via its meshwork structure. This fact was supported by the finding that the diffusion of medium-sized anionic LP from the injection site exhibited more spreading pattern as the result of the HAase treatment (Figure S12). Regarding the leakage of the LPs to the blood circulation, two pathways are possible: direct leakage through the blood vessel around the injection site, or via the subclavian vein after the passage through the lymphatic trafficking through ILNs and ALNs. If the former pathway were true, the non-specific degradation of ECM would be expected to non-specifically enhance the hepatic accumulation of LPs regardless of the surface charge. However, the hepatic accumulation of anionic LPs was not changed by HAase treatment. These collective data imply that the leakage of LPs to the blood circulation and hepatic accumulation dominantly occurred via the subclavian vein after lymphatic transport. In contrast with the primary LNs, the uptake of the neutral and anionic LPs in each macrophage fraction in the secondary LN was not significantly different (Figure 6B; Figure S8). This can be attributed to the lowered accumulation of anionic LPs in the secondary LN compared with the primary LN (Figure 2B). In fact, the MSM uptake of anionic LPs was slightly lower than the values for the neutral ones. In other words, the difference in the cell population that captures anionic and neutral LPs might not be attributed to functional or morphological differences between ILNs and ALNs. Further experiments will be needed to elucidate whether LNs in different sites are identical or not.

Physiologically, the SSMs and MSMs are involved in the uptake of virus particles and the presentation of antigens to B cells,[32] suggesting that LPs likewise would be captured by these cells. Although little is known regarding the MCMs, subcutaneously injected soluble antigens were reported to be taken up by the SSMs and MSMs, but not the MCMs.[33] Some research suggests that the MCM frequently contains intrinsic apoptotic plasma cells.[34,35] Because MCMs are a specialized cell for capturing intrinsic plasma cells, but not extrinsic particles, the MCMs might take up lower levels of LPs than others. The preferred route for the uptake of anionic LPs to the SSMs and MSMs in preference to the MCMs *in vivo* can be explained by the histology of the LN: the SSM and MSM are preferentially exposed to the fluid flow because they are in the subcapsular medullary sinus, which is close to lymphatic flow. Actually, SSMs and MSMs are specialized cells that function to capture extrinsic antigens and materials in the lymphatic sinuses. LPs in the lymphatic sinuses would be recognized and taken up by these cells. In contrast, MCM plays a unique role in intrinsic apoptotic cells and is not considered to be involved in the phagocytosis of antigens in the sinuses. The exact mechanism for the anionic LP-preferred uptake against neutral ones continues to remain to be elucidated.

Anionic LPs are bound to macrophages by energy-independent absorption (Figure S10), perhaps because of receptors such as scavenger receptors.[36] Because the binding assay was carried out in the absence of serum proteins, the surface charge per se might be responsible for

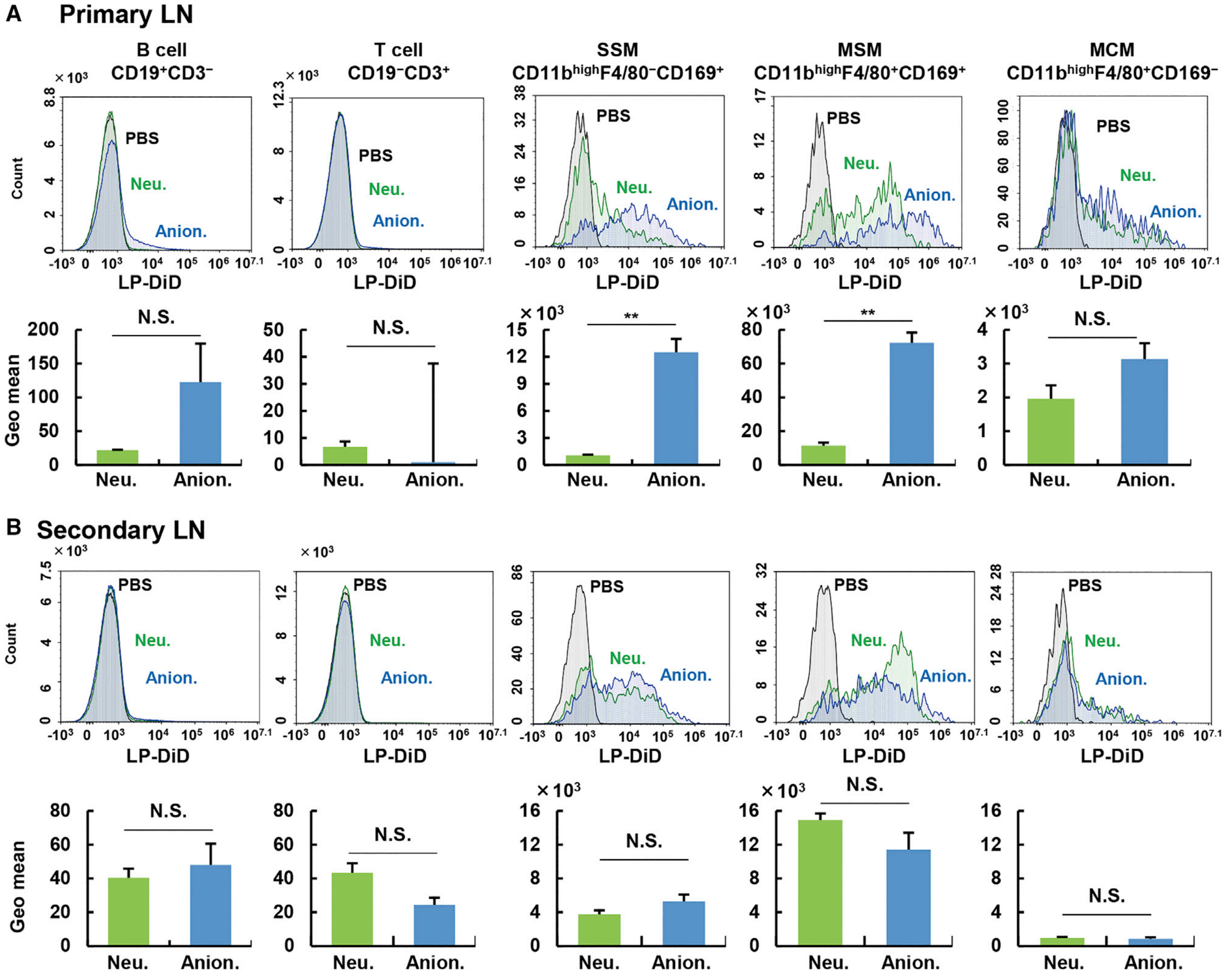

Figure 6. The Distribution of Each LP in the Primary and Secondary LNs

(A and B) Contribution to the uptake of LPs by each cell population in (A) primary LNs and (B) secondary LNs. Cellular uptake of subcapsular sinus macrophages (SSMs; $CD11b^{high}F4/80^{-}CD169^{+}$), medullary sinus macrophages (MSMs; $CD11b^{high}F4/80^{+}CD169^{+}$), medullary cord macrophages (MCMs; $CD11b^{high}F4/80^{+}CD169^{-}$), B cells ($CD19^{+}CD3^{-}$), and T cells ($CD19^{-}CD3^{+}$) was evaluated at 24 h after the injection. The value represents the mean ± SE. For pairwise comparison, Student's t test was performed. $^{**}p < 0.01$. When the p value is over 0.05, the difference is regarded not statistically significant.

binding of anionic LPs to macrophages rather than proteins bound to LPs in response to the surface charge. This receptor-mediated uptake of anionic LPs in the SSMs and MSMs is also supported by the finding that the retention of the anionic LPs in primary LNs was canceled when higher doses of the anionic LPs were administered (Figure S13). In this case, the receptor is saturated, and the anionic LPs that avoid phagocytosis by the SSMs and MSMs were drained into the secondary LNs. Unexpectedly, when the uptakes of small and large anionic LPs by the SSMs, MSMs, and MCMs were compared with same assay system using the LN cell suspension, the small-sized particles were taken up by these cells at essentially the same levels in comparison with middle LPs (Figure S14). Thus, the extensive *in vivo* retention of medium-sized anionic LPs cannot be explained by its preferred uptake by the SSMs and MSMs. Although the mechanism remains to be clarified, it is possible that the small-sized LPs flow through the lymphatic fluid more rapidly, and thus the exposure time to the SSMs and MSMs is shorter. Actually, low-intensity signals in the intermediate sinus and medullary sinus were detected after the administration of small-sized anionic LPs (Figure S15). Collectively, anatomically different locations of these macrophages appear to determine the extent of anionic LPs uptake by each macrophage.

Because the subcutaneous injection of HAase is currently applied for an increase of injection volume of antibody drug in clinic,[37] co-injection of LPs and HAase in a surgical operation could be expected to be clinically reliable procedure. In other words, HAase treatment can be used in the medical application without severe adverse effects. We concluded that a co-injection of fluorescent medium-sized anionic

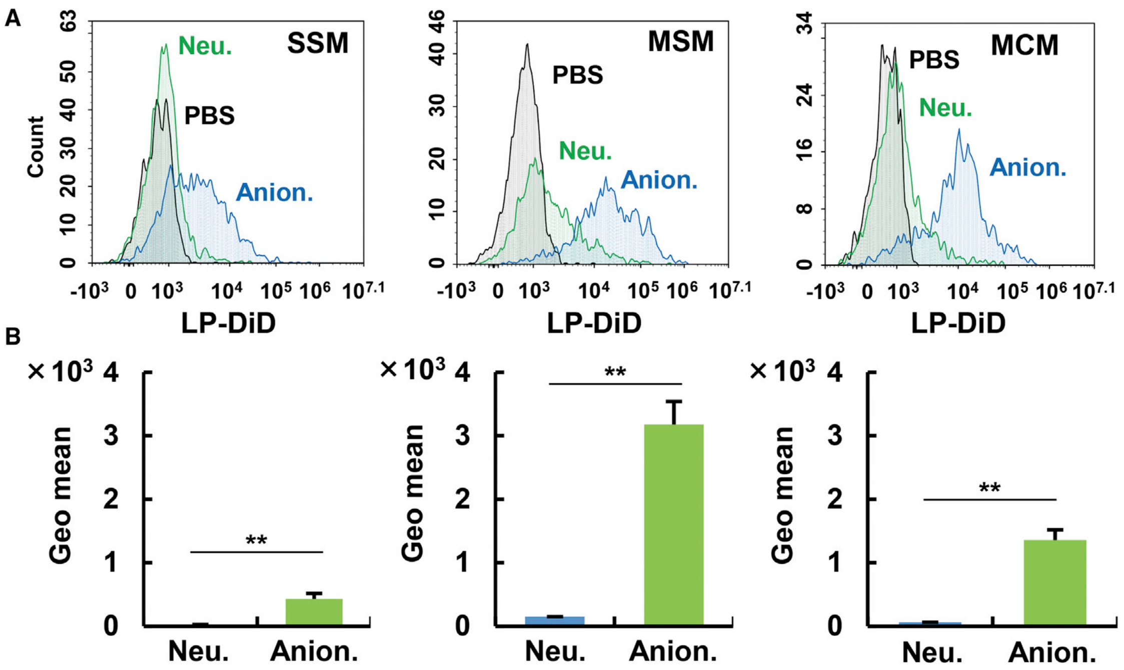

Figure 7. Evaluation of the Affinity between Macrophages and LPs Using Cell Suspensions

LPs were incubated with cell suspensions extracted from LNs from naive mice for 2 h. The cells were then analyzed by flow cytometry. (A) Upper histograms are representative data. (B) Geometrical means were calculated from independent experiments. The value represents the mean ± SE (n = 3). Student's t test was performed for comparison between neutral and anionic LPs. **p < 0.01.

LP and HAase was a promising strategy for the selective imaging of sentinel LNs.

Conclusions

A comprehensive analysis on intra-lymphatic system kinetics of LPs with different physicochemical properties revealed that mid-sized (approx. 130 nm) anionic LPs preferred to accumulate in primary LNs after subcutaneous injection. This preferable distribution of anionic LPs allows for sentinel LN imaging with a high sensitivity in comparison with ICG and other LPs. In addition, the degradation of HA by HAase significantly stimulated immigration into the lymphatic system from the subcutis, and therefore enabled nanoparticles to more clearly detect the sentinel LN. Fluorescence-labeled medium-sized anionic LPs in combination with HAase would be a potent strategy to allow for intra-operative biopsies via the specific, bright detection of sentinel LNs.

MATERIALS AND METHODS

Materials

Egg phosphocholine (EPC) and poly(ethyleneglycol)-dimyristoyl-*sn*-glycerol (PEG-DMG) were obtained from the NOF Corporation (Tokyo, Japan). chol was purchased from Sigma-Aldrich (St. Louis, MO, USA). CHEMS, 3β-[N-(N′,N′-dimethylaminoethane)-carbamoyl], and DC-chol were purchased from Avanti Polar Lipids (Alabaster, AL, USA). DiI (2-[3-(1,3-Dihydro-3,3-dimethyl-1-octadecyl-2H-indol-2-ylidene)-1-propenyl]-3,3-dimethyl-1-octadecyl-3H-indolium perchlorate), DiD, and DiR (2-[7-(1,3-Dihydro-3,3-dimethyl-1-octadecyl-2H-indol-2-ylidene)-1,3,5-heptatrien-1-yl]-3,3-dimethyl-1-octadecyl-3H-indolium perchlorate) were obtained from Biotium (Fremont, CA, USA). HAase from bovine testes and phosphate-buffered saline without Ca^{2+} and Mg^{2+} (PBS) were purchased from Nacalai Tesque (Kyoto, Japan). 2-[4-(2-Hydroxyethyl)piperazin-1-yl]ethanesulfonic acid (HEPES) was obtained from Dojindo (Kumamoto, Japan). Antibodies were listed in Figure S16.

Preparation of LPs

LPs were prepared by the thin-layer hydration method. EPC and chol or anionic and cationic derivatives (CHEMS and DC-chol) in ethanol were added to glass tubes at a 50/50 molar ratio. PEG-DMG was added to the solution at 1 mol % of the total lipid. Ethanol was removed *in vacuo*. Chloroform was added to the lipid residue, and the chloroform was then removed by a stream of dry N_2 gas. The obtained lipid thin layer was hydrated by PBS for 30 min and then sonicated. The mixture was subjected to an Extruder (Avanti Polar Lipids) with a polycarbonate filter (GE Healthcare Japan, Tokyo, Japan). Filters with 50-, 100-, or 400-nm pores were used. For fluorescent labeling, DiI, DiD, or DiR was added to the lipid mixture at 0.5, 0.1, and 0.2 mol %, respectively. Before the LPs were used in experiments, the fluorescent intensities of each sample were measured using an M200 plate reader (TECAN, Männedorf, Switzerland). The fluorescent intensities were then adjusted to the same among all samples. The characterization of the LPs was carried out with a Zetasizer Nano

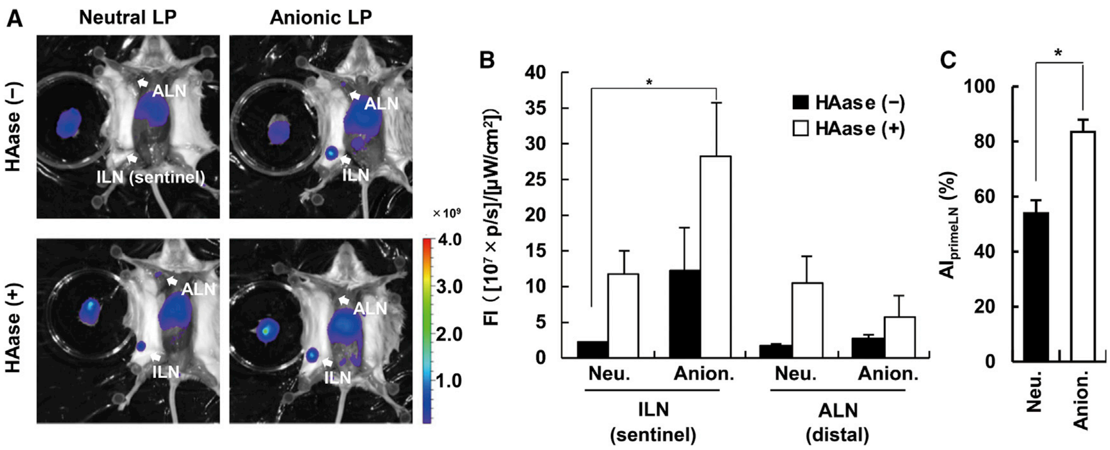

Figure 8. Improved Method for Imaging Sentinel LN in a Breast Cancer Orthotopic Model

Mice were sacrificed at 6 h after the intratumoral injection of LPs and HAase treatment at the same time. (A) Representative images of each sample are shown. (B) Quantitative analysis from the images. The value represents the mean ± SE (n = 3–5). To assess the accumulation driven by HAase in the same LN group, we performed non-repeated ANOVA, followed by Student-Newman-Keuls test. *$p < 0.05$. (C) $AI_{primeLNs}$ of neutral and anionic LPs were compared. The Student's t test was performed for statistical analysis. *$p < 0.05$.

ZS (Malvern Instruments, Malvern, UK). For ζ-potential evaluation, LP solution was dispersed in 10 mM HEPES buffer (pH 7.4). The diameters of LPs were measured in PBS.

Manipulation of the LFM Model

The LFM model was prepared according to our previous report.[16] In brief, a small aliquot of 2% Patent Blue was subcutaneously administered into the right foot pad of mice under isoflurane anesthesia to visualize the lymphatic vessels and the popliteal LNs. The lymphatic and blood vessels were ligated by a nylon thread, and the popliteal LN subsequently resected. The experiments on the intra-lymphatic system distribution of LPs were carried out 7 days after the surgery.

Preparation of Murine Breast Cancer Orthotopic Model

4T1 cells were maintained in RPMI 1640 with 10% fetal bovine serum, 100 U/mL penicillin, and 100 μg/mL streptomycin at 37°C in 5% CO_2 atmosphere. Under isoflurane anesthesia, 5.0×10^5 cells were subcutaneously administered into the mammary fat pad. The imaging of sentinel LNs was carried out approx. 3 weeks after the inoculation. LPs were administered into the tumor tissue at 250 nmol lipid with or without 2,500 U HAase. All animal experiments were approved by the Institutional Animal Care and Use Committee of Chiba University.

Evaluation of Intra-lymphatic System Distribution of LPs

DiR-labeled LPs were subcutaneously injected into the footpad of LFM mice at 100 nmol/20 μL/mouse. As mentioned above, the fluorescent intensity of each of the LPs was adjusted by diluting samples with PBS. Although the actual dosages of lipid were slightly decreased by this dilution (0.67- to 0.81-fold lower than the LP with maximum fluorescence; Figure S17), the results shown in Figure S12 suggest that the slight difference in the lipid dosage (at a maximum 1.5-fold) had no effect on the intra-lymphatic kinetics of LPs. At the indicated times, the LPs in the ILN and ALN were observed by IVIS Lumina II (imaging mode: fluorescent; exposure time: 1 s; binning: medium; lamp level: high; excitation filter: 745 nm; emission filter: ICG) under isoflurane anesthesia.

Observation of the Distribution of LPs in LNs

To investigate the localization of LPs in LNs, we administered DiI-labeled LPs into the tail base of normal mice. LNs were collected at 24 h after the injection and then fixed in 4% paraformaldehyde for 1 h. The fixed LNs were embedded in optical cutting temperature (OCT) compound (Sakura Finetek Japan, Tokyo, Japan). The embedded tissues were sliced at a thickness of 8 μm. The slices were immersed in a solution of a CD11b rat antibody (clone: M1/70; BioLegend) 200 times diluted with 1.5% bovine serum albumin (BSA) in PBS. They were then stained in 1 μg/mL Hoechst 33342 (Sigma-Aldrich) and Alexa Fluor 647 goat anti-rat IgG (Thermo Fisher Science). The slices were covered with coverslips (No. 1S) with Prolong Diamond (Thermo Fisher Science) and then observed by a BZ-X710 system (Keyence, Tokyo, Japan).

Determination of Cell Population Taking in LPs in LNs Using Flow Cytometry

DiD-labeled LPs were injected into the foot pad of the LFM model. The ILNs and ALNs were resected at 24 h after the administration. LNs were digested in 0.1 mg/mL collagenase IV, 0.2 mg/mL collagenase D, and 0.1 mg/mL DNase I in RPMI 1640 (with 1 v/v % FBS) for 30 min. These were reliable procedures performed in the previous several reports.[38–40] The cell suspensions were washed twice with 0.5% BSA/0.1% sodium azide in PBS (fluorescence-activated cell sorting [FACS] buffer). After dispersing the cell suspensions with a 70-μm cell strainer, 2.0×10^6 cells were incubated in 10 μg/μL of anti-mouse CD16/32 antibody (clone: 93; BioLegend). Cells were stained

and analyzed with a Novocyte (ACEA Biosciences, San Diego, CA, USA) according to Figure S7.

SUPPLEMENTAL INFORMATION

Supplemental Information can be found online at https://doi.org/10.1016/j.ymthe.2020.09.014.

AUTHOR CONTRIBUTIONS

The manuscript was written through contributions of all of the authors. All of the authors have given approval to the final version of the manuscript. M.G. and N.M. performed all of the experiments. Y.S., H.T., and H.A. contributed to the experimental design and hypothesis of scientific theme. T.A. performed intravital imaging.

CONFLICTS OF INTEREST

The authors declare no competing interests.

ACKNOWLEDGMENTS

The authors wish to thank Milton Feather for the English correction of this manuscript. The research described in this manuscript was supported, in part, by grants from the Japan Society for the Promotion of Science KAKENHI (grants 18K18351 and 20K20195), JST CREST, Japan (grant JPMJCR17H1), the Mochida Memorial Foundation for Medical and Pharmaceutical Research, and the Ichiro Kanehara Foundation.

REFERENCES

1. Sainte-Marie, G. (2010). The lymph node revisited: development, morphology, functioning, and role in triggering primary immune responses. Anat. Rec. (Hoboken) *293*, 320–337.
2. Girard, J.P., Moussion, C., and Förster, R. (2012). HEVs, lymphatics and homeostatic immune cell trafficking in lymph nodes. Nat. Rev. Immunol. *12*, 762–773.
3. Doepker, M.P., and Zager, J.S. (2015). Sentinel lymph node mapping in melanoma in the twenty-first century. Surg. Oncol. Clin. N. Am. *24*, 249–260.
4. Reintgen, M., Kerivan, L., Reintgen, E., Swaninathan, S., and Reintgen, D. (2016). Breast Lymphatic Mapping and Sentinel Lymph Node Biopsy: State of the Art: 2015. Clin. Breast Cancer *16*, 155–165.
5. Matsuura, Y., Ichinose, J., Nakao, M., Okumura, S., and Mun, M. (2019). Recent fluorescence imaging technology applications of indocyanine green in general thoracic surgery. Surg. Today. Published online October 29, 2019. https://doi.org/10.1007/s00595-019-01906-6.
6. Goyal, A. (2018). New Technologies for Sentinel Lymph Node Detection. Breast Care (Basel) *13*, 349–353.
7. Ahmed, M., Purushotham, A.D., and Douek, M. (2014). Novel techniques for sentinel lymph node biopsy in breast cancer: a systematic review. Lancet Oncol. *15*, e351–e362.
8. Sugie, T., Sawada, T., Tagaya, N., Kinoshita, T., Yamagami, K., Suwa, H., Ikeda, T., Yoshimura, K., Niimi, M., Shimizu, A., and Toi, M. (2013). Comparison of the indocyanine green fluorescence and blue dye methods in detection of sentinel lymph nodes in early-stage breast cancer. Ann. Surg. Oncol. *20*, 2213–2218.
9. Verbeek, F.P., Troyan, S.L., Mieog, J.S., Liefers, G.J., Moffitt, L.A., Rosenberg, M., Hirshfield-Bartek, J., Gioux, S., van de Velde, C.J., Vahrmeijer, A.L., and Frangioni, J.V. (2014). Near-infrared fluorescence sentinel lymph node mapping in breast cancer: a multicenter experience. Breast Cancer Res. Treat. *143*, 333–342.
10. Tagaya, N., Yamazaki, R., Nakagawa, A., Abe, A., Hamada, K., Kubota, K., and Oyama, T. (2008). Intraoperative identification of sentinel lymph nodes by near-infrared fluorescence imaging in patients with breast cancer. Am. J. Surg. *195*, 850–853.
11. Takemoto, N., Koyanagi, A., Yasuda, M., and Yamamoto, H. (2018). Comparison of the indocyanine green dye method versus the combined method of indigo carmine blue dye with indocyanine green fluorescence imaging for sentinel lymph node biopsy in breast conservative therapy for stage ≤IIA breast cancer. BMC Womens Health *18*, 151.
12. Trevaskis, N.L., Kaminskas, L.M., and Porter, C.J. (2015). From sewer to saviour - targeting the lymphatic system to promote drug exposure and activity. Nat. Rev. Drug Discov. *14*, 781–803.
13. Rohner, N.A., and Thomas, S.N. (2017). Flexible Macromolecule versus Rigid Particle Retention in the Injected Skin and Accumulation in Draining Lymph Nodes Are Differentially Influenced by Hydrodynamic Size. ACS Biomater. Sci. Eng. *3*, 153–159.
14. Reddy, S.T., Berk, D.A., Jain, R.K., and Swartz, M.A. (2006). A sensitive in vivo model for quantifying interstitial convective transport of injected macromolecules and nanoparticles. J. Appl. Physiol. (1985) *101*, 1162–1169.
15. Kaminskas, L.M., Kota, J., McLeod, V.M., Kelly, B.D., Karellas, P., and Porter, C.J. (2009). PEGylation of polylysine dendrimers improves absorption and lymphatic targeting following SC administration in rats. J. Control. Release *140*, 108–116.
16. Yamaji, Y., Akita, S., Akita, H., Miura, N., Gomi, M., Manabe, I., Kubota, Y., and Mitsukawa, N. (2018). Development of a mouse model for the visual and quantitative assessment of lymphatic trafficking and function by in vivo imaging. Sci. Rep. *8*, 5921.
17. Oussoren, C., Zuidema, J., Crommelin, D.J., and Storm, G. (1997). Lymphatic uptake and biodistribution of liposomes after subcutaneous injection. II. Influence of liposomal size, lipid compostion and lipid dose. Biochim. Biophys. Acta *1328*, 261–272.
18. Rao, D.A., Forrest, M.L., Alani, A.W., Kwon, G.S., and Robinson, J.R. (2010). Biodegradable PLGA based nanoparticles for sustained regional lymphatic drug delivery. J. Pharm. Sci. *99*, 2018–2031.
19. Ali Khan, A., Mudassir, J., Mohtar, N., and Darwis, Y. (2013). Advanced drug delivery to the lymphatic system: lipid-based nanoformulations. Int. J. Nanomedicine *8*, 2733–2744.
20. Müllegger, J., and Lepperdinger, G. (2002). Hyaluronan is an abundant constituent of the extracellular matrix of Xenopus embryos. Mol. Reprod. Dev. *61*, 312–316.
21. Oussoren, C., Velinova, M., Scherphof, G., van der Want, J.J., van Rooijen, N., and Storm, G. (1998). Lymphatic uptake and biodistribution of liposomes after subcutaneous injection. IV. Fate of liposomes in regional lymph nodes. Biochim. Biophys. Acta *1370*, 259–272.
22. Rigotti, A., Acton, S.L., and Krieger, M. (1995). The class B scavenger receptors SR-BI and CD36 are receptors for anionic phospholipids. J. Biol. Chem. *270*, 16221–16224.
23. Guillaume, D., Bertrand, P., Dea, D., Davignon, J., and Poirier, J. (1996). Apolipoprotein E and low-density lipoprotein binding and internalization in primary cultures of rat astrocytes: isoform-specific alterations. J. Neurochem. *66*, 2410–2418.
24. Akita, H., Noguchi, Y., Hatakeyama, H., Sato, Y., Tange, K., Nakai, Y., and Harashima, H. (2015). Molecular Tuning of a Vitamin E-Scaffold pH-Sensitive and Reductive Cleavable Lipid-like Material for Accelerated in Vivo Hepatic siRNA Delivery. ACS Biomater. Sci. Eng. *1*, 834–844.
25. Peng, J., Yang, Q., Shi, K., Xiao, Y., Wei, X., and Qian, Z. (2019). Intratumoral fate of functional nanoparticles in response to microenvironment factor: Implications on cancer diagnosis and therapy. Adv. Drug Deliv. Rev. *143*, 37–67.
26. Lin, G., Chen, S., and Mi, P. (2018). Nanoparticles Targeting and Remodeling Tumor Microenvironment for Cancer Theranostics. J. Biomed. Nanotechnol. *14*, 1189–1207.
27. Chauhan, V.P., Martin, J.D., Liu, H., Lacorre, D.A., Jain, S.R., Kozin, S.V., Stylianopoulos, T., Mousa, A.S., Han, X., Adstamongkonkul, P., et al. (2013). Angiotensin inhibition enhances drug delivery and potentiates chemotherapy by decompressing tumour blood vessels. Nat. Commun. *4*, 2516.
28. Chauhan, V.P., Boucher, Y., Ferrone, C.R., Roberge, S., Martin, J.D., Stylianopoulos, T., Bardeesy, N., DePinho, R.A., Padera, T.P., Munn, L.L., and Jain, R.K. (2014). Compression of pancreatic tumor blood vessels by hyaluronan is caused by solid stress and not interstitial fluid pressure. Cancer Cell *26*, 14–15.
29. van den Berg, J.H., Oosterhuis, K., Hennink, W.E., Storm, G., van der Aa, L.J., Engbersen, J.F., Haanen, J.B., Beijnen, J.H., Schumacher, T.N., and Nuijen, B. (2010). Shielding the cationic charge of nanoparticle-formulated dermal DNA vaccines is essential for antigen expression and immunogenicity. J. Control. Release *141*, 234–240.

30. Zhuang, Y., Ma, Y., Wang, C., Hai, L., Yan, C., Zhang, Y., Liu, F., and Cai, L. (2012). PEGylated cationic liposomes robustly augment vaccine-induced immune responses: Role of lymphatic trafficking and biodistribution. J. Control. Release *159*, 135–142.

31. Nakamura, T., Kawai, M., Sato, Y., Maeki, M., Tokeshi, M., and Harashima, H. (2020). The Effect of Size and Charge of Lipid Nanoparticles Prepared by Microfluidic Mixing on Their Lymph Node Transitivity and Distribution. Mol. Pharm. *17*, 944–953.

32. Junt, T., Moseman, E.A., Iannacone, M., Massberg, S., Lang, P.A., Boes, M., Fink, K., Henrickson, S.E., Shayakhmetov, D.M., Di Paolo, N.C., et al. (2007). Subcapsular sinus macrophages in lymph nodes clear lymph-borne viruses and present them to antiviral B cells. Nature *450*, 110–114.

33. Gray, E.E., and Cyster, J.G. (2012). Lymph node macrophages. J. Innate Immun. *4*, 424–436.

34. Nossal, G.J., Abbot, A., and Mitchell, J. (1968). Antigens in immunity. XIV. Electron microscopic radioautographic studies of antigen capture in the lymph node medulla. J. Exp. Med. *127*, 263–276.

35. Steer, H.W., and Foot, R.A. (1987). Changes in the medulla of the parathymic lymph nodes of the rat during acute gastro-intestinal inflammation. J. Anat. *152*, 23–36.

36. Nishikawa, K., Arai, H., and Inoue, K. (1990). Scavenger receptor-mediated uptake and metabolism of lipid vesicles containing acidic phospholipids by mouse peritoneal macrophages. J. Biol. Chem. *265*, 5226–5231.

37. Wasserman, R.L., Lumry, W., Harris, J., 3rd, Levy, R., Stein, M., Forbes, L., Cunningham-Rundles, C., Melamed, I., Kobayashi, A.L., Du, W., and Kobayashi, R. (2016). Efficacy, Safety, and Pharmacokinetics of a New 10 % Liquid Intravenous Immunoglobulin Containing High Titer Neutralizing Antibody to RSV and Other Respiratory Viruses in Subjects with Primary Immunodeficiency Disease. J. Clin. Immunol. *36*, 590–599.

38. Camara, A., Cordeiro, O.G., Alloush, F., Sponsel, J., Chypre, M., Onder, L., Asano, K., Tanaka, M., Yagita, H., Ludewig, B., et al. (2019). Lymph Node Mesenchymal and Endothelial Stromal Cells Cooperate via the RANK-RANKL Cytokine Axis to Shape the Sinusoidal Macrophage Niche. Immunity *50*, 1467–1481.e6.

39. Asano, K., Nabeyama, A., Miyake, Y., Qiu, C.H., Kurita, A., Tomura, M., Kanagawa, O., Fujii, S., and Tanaka, M. (2011). CD169-positive macrophages dominate antitumor immunity by crosspresenting dead cell-associated antigens. Immunity *34*, 85–95.

40. Phan, T.G., Green, J.A., Gray, E.E., Xu, Y., and Cyster, J.G. (2009). Immune complex relay by subcapsular sinus macrophages and noncognate B cells drives antibody affinity maturation. Nat. Immunol. *10*, 786–793.

YMTHE, Volume *29*

Supplemental Information

Development of Sentinel LN Imaging with a Combination of HAase Based on a Comprehensive Analysis of the Intra-lymphatic Kinetics of LPs

Masaki Gomi, Yu Sakurai, Takaharu Okada, Naoya Miura, Hiroki Tanaka, and Hidetaka Akita

Supplemental Information

Development of sentinel lymph node imaging with a combination of hyaluronidase based on a comprehensive analysis of the intra-lymphatic kinetics of liposomes

Masaki Gomi[1,‡], Yu Sakurai[1,‡,*], Takaharu Okada[2], Naoya Miura[1], Hiroki Tanaka[1], Hidetaka Akita[1,*].

AUTHOR ADDRESS

[1] Graduate School of Pharmaceutical Sciences, Chiba University, Chiba, Japan.

[2] RIKEN Center for Integrative Medical Sciences, Yokohama, Kanagawa, Japan

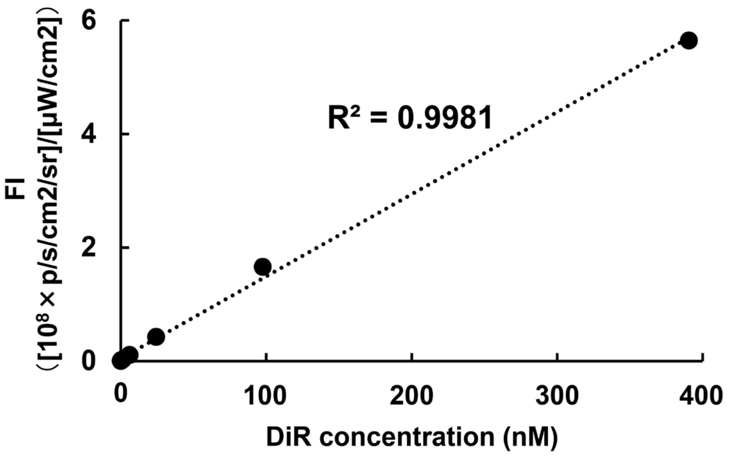

Supplemental Figure 1. Confirmation of the linearity of DiR fluorescence. The fluorescent intensity of known concentrations of DiR in the solution was detected by IVIS.

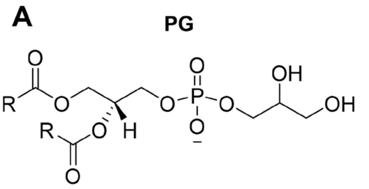

	Z-Ave. (nm)	PdI	ζ-potential (mV)
PG	122±1	0.08±0.03	-24±1

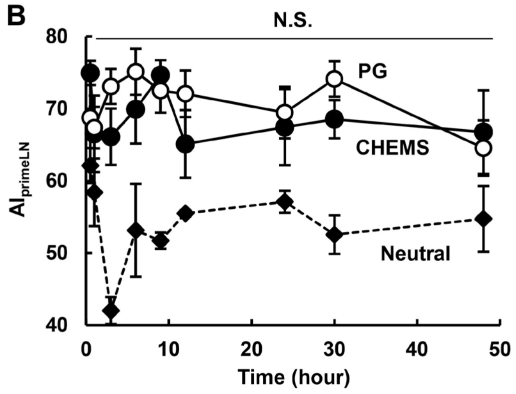

Supplemental Figure 2. The intra-lymphatic system distribution of LPs with other anionic lipids. A) The structure of PG is shown. R means -$C_{18}H_{33}$. LPs were characterized by DLS. B) Percentage of remaining in the primary LN was evaluated for periods of up to 48 hours. Data represents the mean ± SE (n=3). Student's t-test was carried out between PG and CHEMS LP at each time points. P-value > 0.05 was regarded as not statistically significant difference (N.S.).

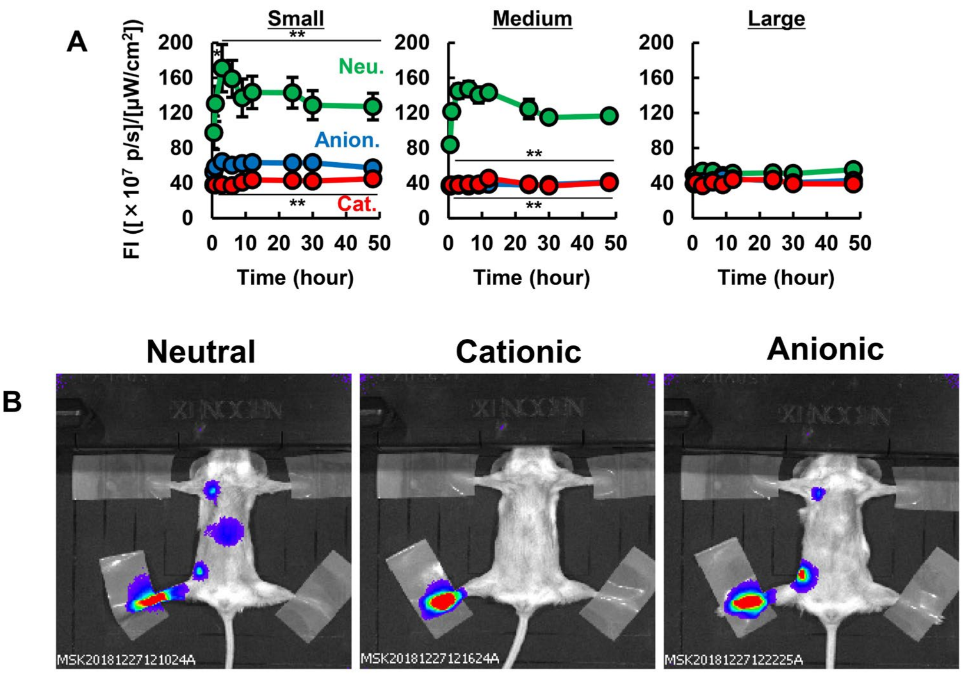

Supplemental Figure 3. Liver accumulation after the administration of LPs into lymphatic system. (A) Transport to the liver after the subcutaneous administration was measured by IVIS. The value represents the mean ± SE (n=3). At each time points, ANOVA was carried out, followed by Bonferroni test versus neutral LPs. N.S.: not statistically significant, *: P<0.05, **: P<0.01. (B) The representative images after the injection of medium-sized neutral, cationic and anionic LPs.

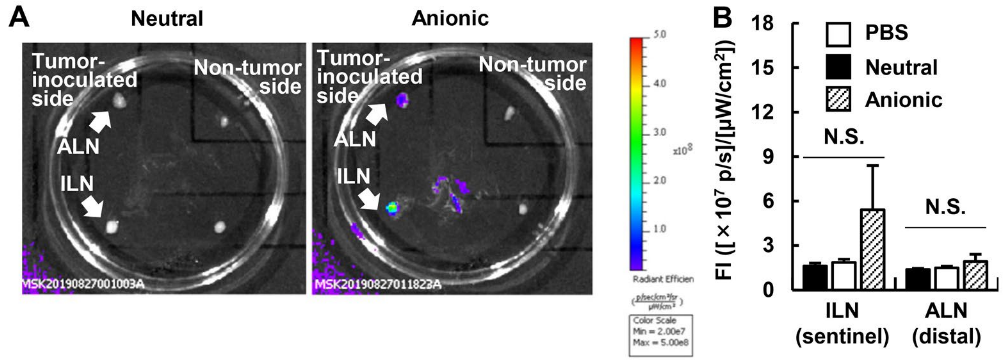

Supplemental Figure 4. Imaging of sentinel LNs in an orthotopic breast cancer model by medium-sized LPs. (A) Representative images of ILN (regarded as sentinel LN) and ALN (regarded as distal LN) of tumor-inoculated side (left) and not-inoculated side (right) 1 hour after the intratumoral injection of LPs. (B) The quantitative data from the (A) images. The value represents mean ± SE (n=3-5). At each time points, ANOVA was carried out, followed by Bonferroni test. N.S.: not statistically significant.

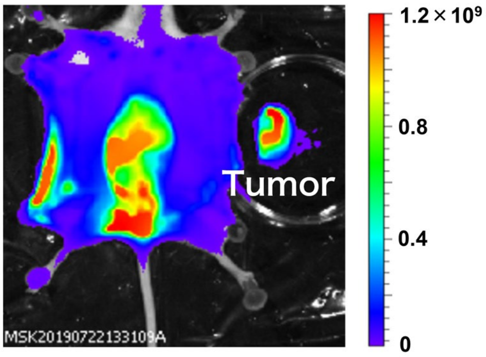

Supplemental Figure 5. The distribution of intratumoral injected indocyanine green (ICG). ICG was visualized by an IVIS system 1 hour after 20 μL of the ICG solution in PBS at 2.5 mg/mL was intratumorally administered.

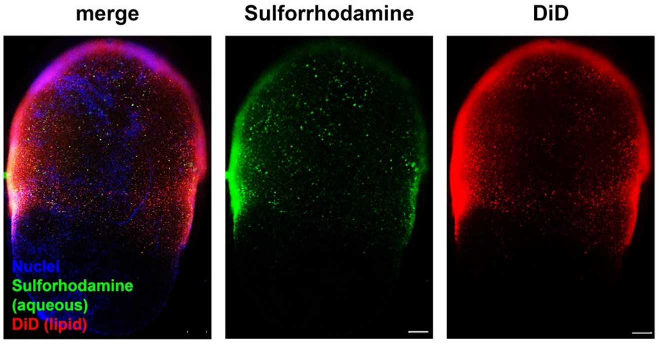

Supplemental Figure 6. Proof of LP delivery in intact form. Sulforhodamine was encapsulated into DiD-labeled anionic LPs. LN was observed 3 hours after the injection of LPs into LFM model. Blue, green and red dots show nuclei, sulforhodamine and DiD, respectively.

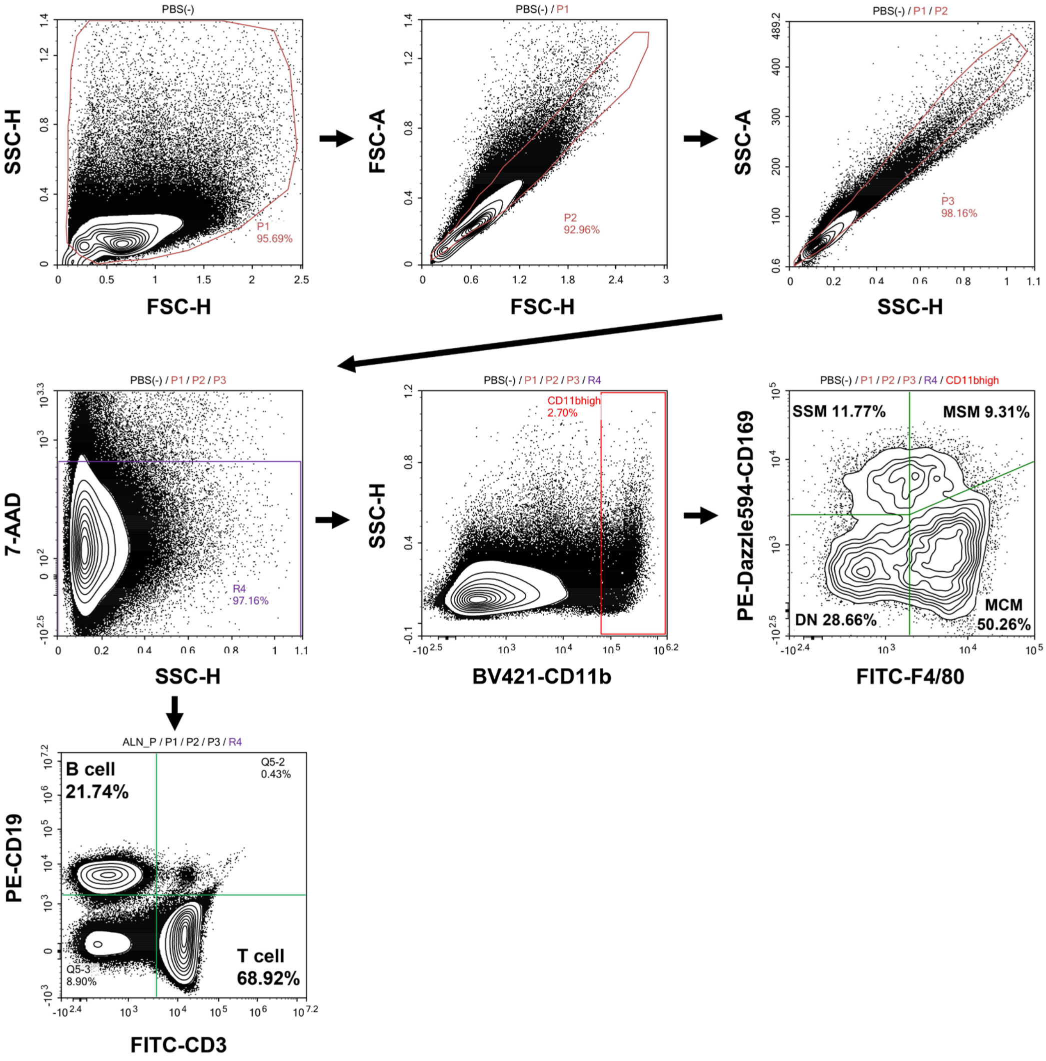

Supplemental Figure 7. A representative flow cytometer plot identifying SSM, MSM and MCM.

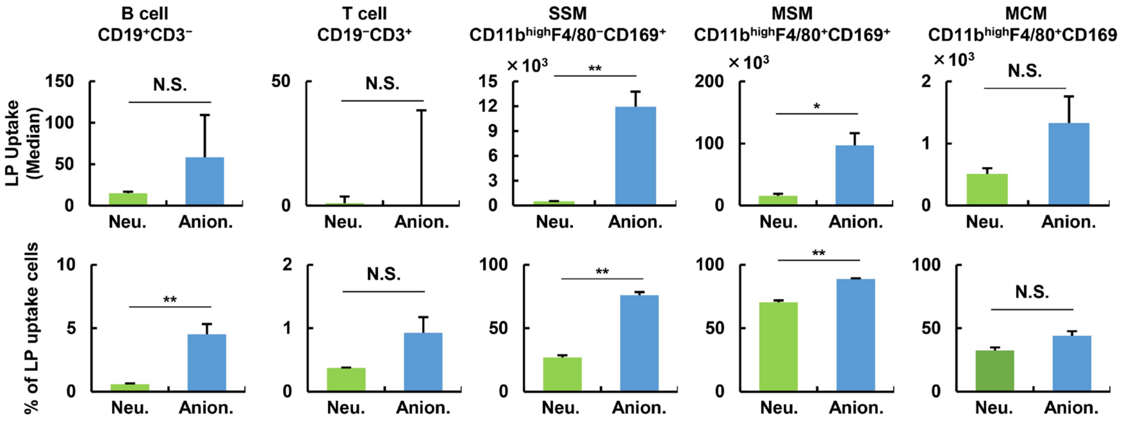

Supplemental Figure 8. Other quantitative data of cells with LPs. Median value of LPs uptake and percentages of the cells taking up LPs were analyzed. Data represent the mean ± SE (n=3). Student's t-test was performed for a pair-wise comparison. *: P<0.05, **: P<0.01.

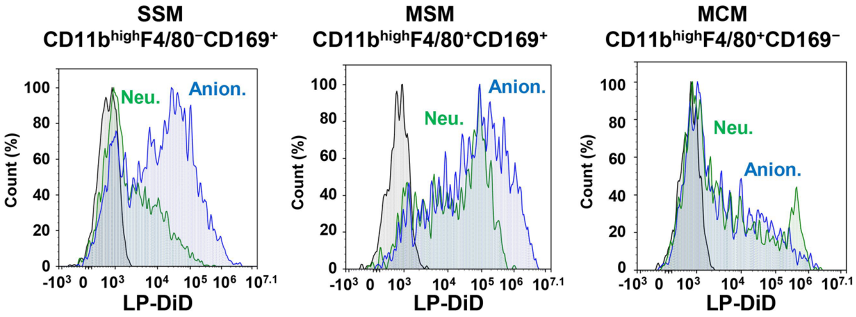

Supplemental Figure 9. The uptake of macrophages with normal (non-operated) mice. LNs were collected 2 hours after the LPs were administered into the tail base (BALC/c mice). Cells with LPs in the ILN were analyzed by flow cytometry.

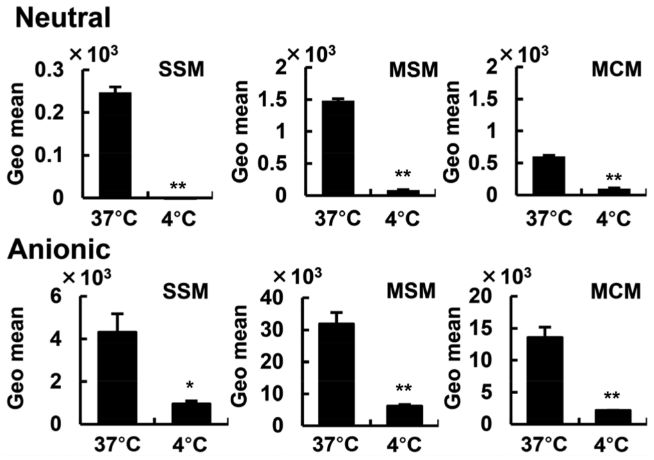

Supplemental Figure 10. The energy-dependent uptake of LPs by macrophages. LPs were incubated with cell suspensions isolated from ILN for 2 hours. Cells were analyzed by flow cytometry. Data represent the mean ± SE (n=3). Student's t-test was performed for a pair-wise comparison. *: P<0.05, **: P<0.01.

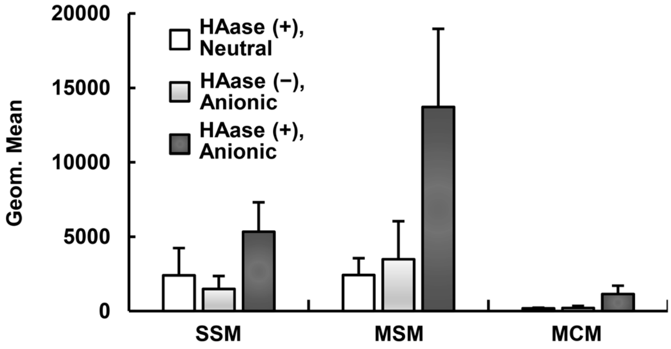

Supplemental Figure 11. Uptake of LPs by macrophages with tumor-bearing mice after intratumoral injection. Sentinel LNs were collected at 6 hours after the intratumoral injection of LPs. LNs were analyzed by flow cytometry. Data represents mean ± SE (n=3).

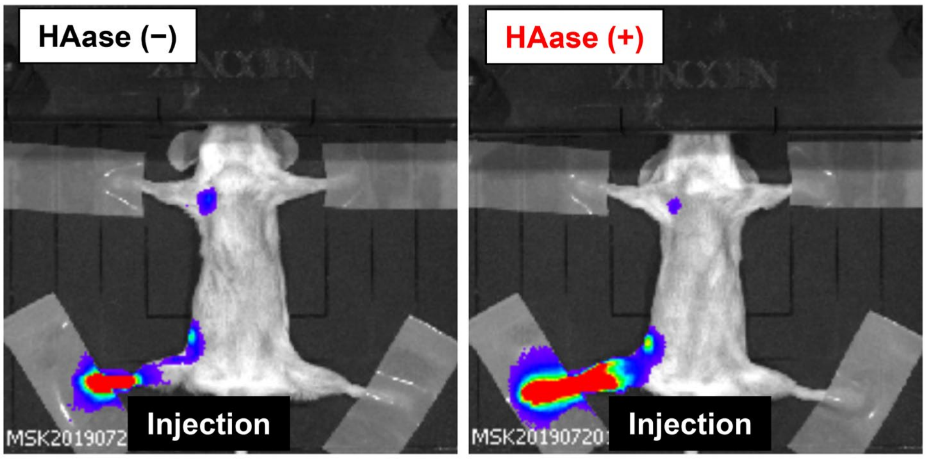

Supplemental Figure 12. The diffusion of LPs after the subcutaneous injection of anionic LPs into LFM mice. Mice were imaged by an IVIS system 30 min the injection.

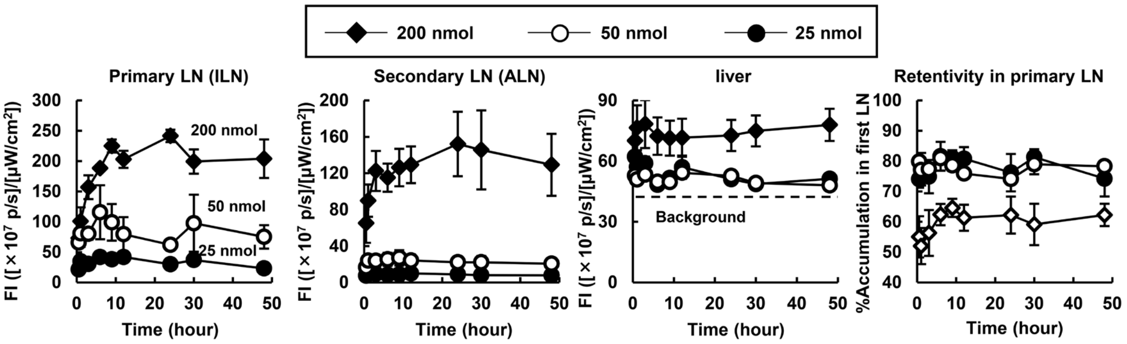

Supplemental Figure 13. Dose-dependent intra-lymphatic system distribution of medium sized anionic LPs. LPs were administered into the foot pad of LFM mice at a concentration of 25 – 200 nmol (100 nmol was used in the manuscript). LPs in the body were observed by an IVIS system for periods of up to 48 hours. Data represent the mean ± SE (n=3).

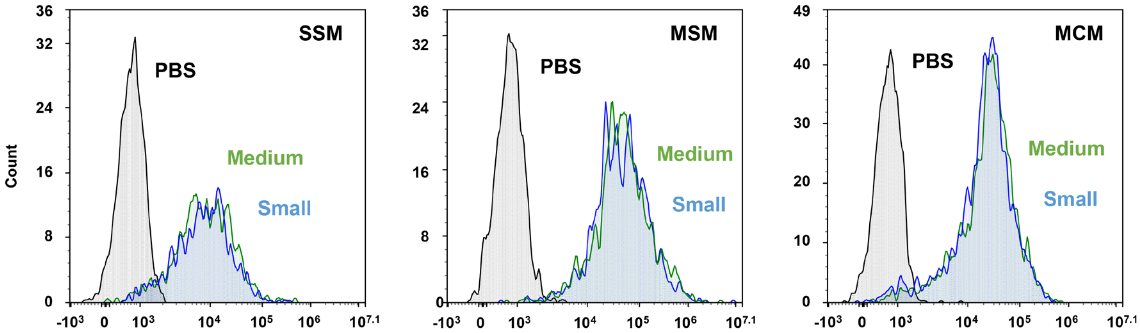

Supplemental Figure 14. The uptake small- and medium sized anionic LPs by macrophages. Small- and Medium-sized anionic LPs were incubated with a cell suspension from the LNs for 2 hours.

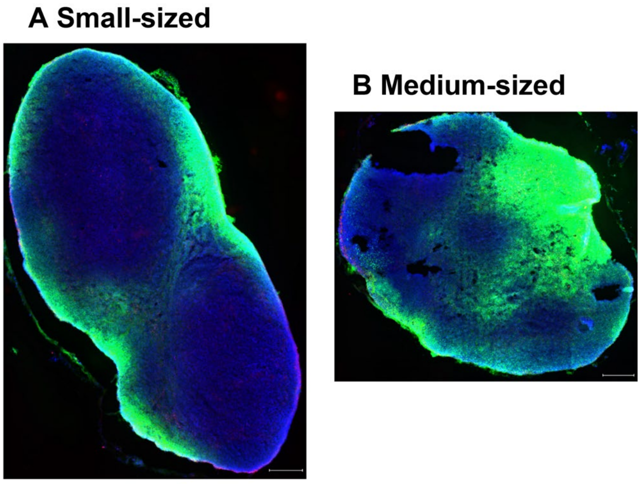

Supplemental Figure 15. The different distribution of anionic LPs with different sizes. A), B) After the subcutaneous injection of A) small-sized and B) medium sized anionic LPs, excised LNs were observed by microscopy 24 hours later. Blue and Green dots denote nuclei (Hoechst33342) and LPs (DiI), respectively.

Supplemental Figure 16. Antibodies and reagents used for the flow cytometry analysis.

Antibody/Reagent	clone/manufacture or catalog No.
CD16/32 FcγR blocking antibody	93
7-AAD	Biolegend, 420404
BV421-CD11b	M1/70
FITC-F4/80	BM8
PE/Dazzle594-CD169	3D6.112
FITC-CD3	17A2
PE-CD19	6D5

A Fluorescent intensity of each LPs

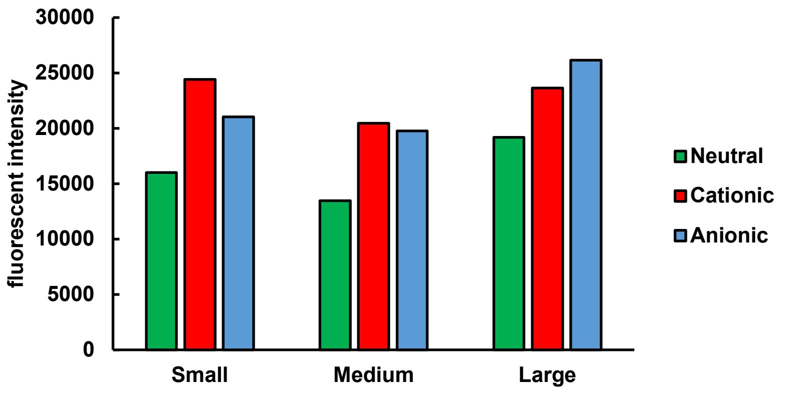

B Phospholipid concentration

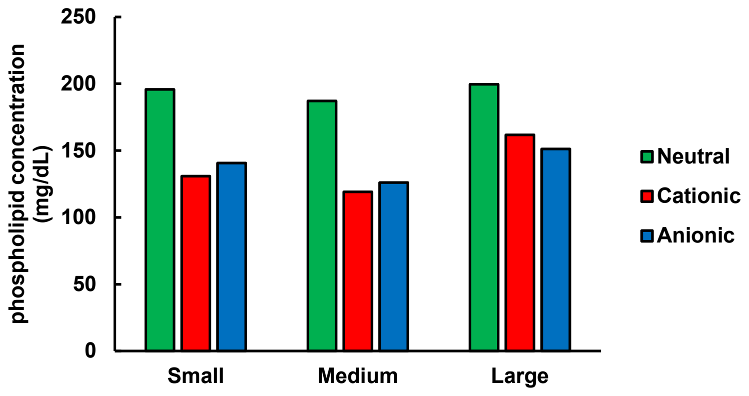

Supplemental Figure 17 The fluorescent intensity and phospholipid concentration of LPs used in Figure 2.

Supplemental materials & methods

Intravital imaging of popliteal LNs.

The entire procedure was performed in reference with the previous report.[1] CD11c-EYFP mice[2] were anesthetized with 1.5% isoflurane (Wako) in a stream of oxygen. Hair on the right hind legs was removed by an electric trimmer and the epilat cream (Kracie), and the epilated skin was cleaned and dried. Surgery was performed according to the previously described method (ref. 2), except that the leg holder assembly was modified as below such that intravital imaging of popliteal LNs could be carried out with an inverted microscope. The leg holder was assembled from a 35-mm Petri dish lid and two Eppendorf tube lids glued on the top of the Petri dish lid. The Eppendorf tube lids were placed approximately 6 mm apart from each other, both near the rim of the Petri dish lid. The right hind leg was glued between the Eppendorf tube lids with Aron alpha A "Sankyo" (Daiichi-Sankyo), with the back of the knee facing up. Skin flaps were made according to the previous report (ref. 2) and glued over the Eppendorf tube lids. The popliteal lymph node was micro-surgically exposed as described previously (ref. 2). The entire preparation was placed on an imaging platform (LEICA MATS, Tokai Hit) adjusted to 37 ℃, with the mouse body lying sideways, the leg holder placed upside down, and the exposed LN touching down on a drop of PBS on a coverslip which was glued with High Silicone II (Denken-Highdental) over the hole of the imaging platform. A catheter (C10SS-MTV1417P, Instech Laboratories) attached to a 1-ml syringe containing Sulforhodamine B-encapsulting LPs (40 nmol/ml lipid conc. in PBS) was inserted into the right hind footpad and immobilized by a vinyl tape. Image stacks of 45 z-sections at a x-y resolution of 0.16μm per pixel with a 1.0μm spacing between neighboring z-sections were acquired every 10 s by using the THUNDER 3D Live Imaging system (Leica Microsystems) equipped with an HC PLAPO CS2 40xW objective lens. EYFP signals were obtained by using a 470/24-nm LED and a 515/40-nm emission filter, and sulforhodamine signals were obtained by using a 550/15-nm LED and a 595/40-nm emission filter. During the time-lapse recordings, 50 μl of the LP suspension was injected into the footpad in 10~20 s. Obtained images were processed by the THUNDER Large Volume Computational Clearing algorithm (Leica Microsystems) using the following settings: Feature Scale 843 nm, Thunder Strength 94%, and Deconvolution Setting Auto.

References

1. Liou, H. L.; Myers, J. T.; Barkauskas, D. S.; Huang, A. Y., Intravital imaging of the mouse popliteal lymph node. *J Vis Exp* **2012,** (60).

2. Lindquist, R. L.; Shakhar, G.; Dudziak, D.; Wardemann, H.; Eisenreich, T.; Dustin, M. L.; Nussenzweig, M. C., Visualizing dendritic cell networks in vivo. *Nat Immunol* **2004,** *5* (12), 1243-50.
